# Nanometer-scale photon confinement in topology-optimized dielectric cavities

**DOI:** 10.1038/s41467-022-33874-w

**Published:** 2022-10-21

**Authors:** Marcus Albrechtsen, Babak Vosoughi Lahijani, Rasmus Ellebæk Christiansen, Vy Thi Hoang Nguyen, Laura Nevenka Casses, Søren Engelberth Hansen, Nicolas Stenger, Ole Sigmund, Henri Jansen, Jesper Mørk, Søren Stobbe

**Affiliations:** 1 DTU Electro, Department of Electrical and Photonics Engineering, Technical University of Denmark, Ørsteds Plads 343, DK-2800 Kgs. Lyngby, Denmark; 2 NanoPhoton—Center for Nanophotonics, Technical University of Denmark, Ørsteds Plads 345A, DK-2800 Kgs. Lyngby, Denmark; 3 Department of Civil and Mechanical Engineering, Technical University of Denmark, Nils Koppels Allé, Building 404, DK-2800 Kgs. Lyngby, Denmark; 4 DTU Nanolab, Technical University of Denmark, Building 347, DK-2800 Kgs. Lyngby, Denmark; 5 Center for Nanostructured Graphene, Technical University of Denmark, Building 345C, DK-2800 Kgs. Lyngby, Denmark

**Keywords:** Sub-wavelength optics, Nanophotonics and plasmonics, Nanocavities

## Abstract

Nanotechnology enables in principle a precise mapping from design to device but relied so far on human intuition and simple optimizations. In nanophotonics, a central question is how to make devices in which the light-matter interaction strength is limited only by materials and nanofabrication. Here, we integrate measured fabrication constraints into topology optimization, aiming for the strongest possible light-matter interaction in a compact silicon membrane, demonstrating an unprecedented photonic nanocavity with a mode volume of *V* ~ 3 × 10^−4^ *λ*^3^, quality factor *Q* ~ 1100, and footprint 4 *λ*^2^ for telecom photons with a *λ* ~ 1550 nm wavelength. We fabricate the cavity, which confines photons inside 8 nm silicon bridges with ultra-high aspect ratios of 30 and use near-field optical measurements to perform the first experimental demonstration of photon confinement to a single hotspot well below the diffraction limit in dielectrics. Our framework intertwines topology optimization with fabrication and thereby initiates a new paradigm of high-performance additive and subtractive manufacturing.

## Abstract (web summary)

Here, the authors integrate measured fabrication constraints in topology optimization to design a highly optimized dielectric nanocavity. The theoretically predicted confinement of light below the diffraction limit is confirmed by near- and far-field spectroscopy.

## Introduction

**Fig. 1:**
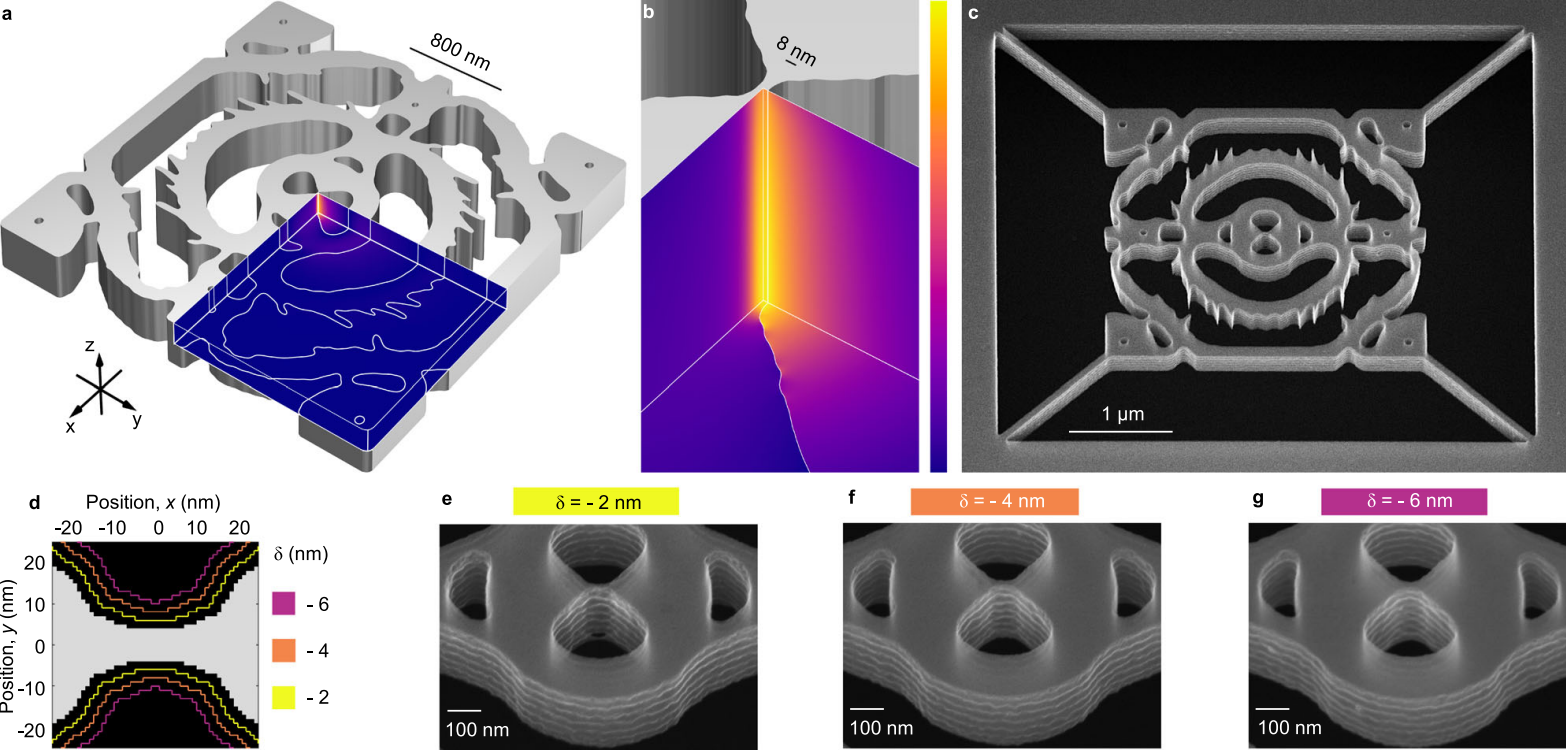
Fabrication of topology-optimized silicon dielectric bowtie cavity (DBC). **a** Rendering of the DBC design generated by tolerance-constrained topology optimization. The normalized ∣**E**∣-field is projected on the faces defining the three symmetry planes of the design. **b** Zoom-in of the solid silicon bowtie exhibiting a strong field confinement due to the bowtie bridge dimension of 8 nm. **c** 40° tilted scanning electron microscopy (SEM) image of a fabricated cavity. **d** Global geometry-tuning, *δ*. Each air (black) pixel (1 nm^2^) inside a *δ*-outline is exposed uniformly with electron-beam lithography; hence, air features defining the device are uniformly tuned. **e**–**g** 40° tilted SEM images of bowtie region for *δ* = { − 2, − 4, − 6} nm. We measure the mean width of the fabricated bowties to be (8 ± 5) nm, (10 ± 5) nm, and (16 ± 5) nm for figures **e**, **f**, and **g**, respectively, noting the variation in width along the *z*-direction caused by the scallops and ~1° negative sidewall angle represented by the uncertainty as discussed in the main text.

Optical nanocavities confine and store light, which is essential to increase the interaction between photons and electrons in semiconductor devices ranging from lasers to emerging quantum technologies^1,2^. A wealth of mechanisms can be exploited for building nanocavities, including distributed Bragg reflection^3–5^, total internal reflection^6^, Fano resonances or bound states in the continuum^7^, and topological confinement^8^. These approaches have achieved orders of magnitude improvements to the temporal confinement, but none of them allow optical mode volumes, *V*, in the deep subwavelength regime. Alternatively, plasmons in metal nanoparticles can confine light below the diffraction limit but the absorption losses in metals^5,9,10^ limit the quality factors to well below 100 (Ref. 11). The approach of our work is entirely different: We also consider loss-less dielectrics but rather than using geometry optimization of designs based on human intuition, we use geometry-agnostic inverse design, i.e., topology optimization^12–14^, to maximize the light-matter interaction in the center of the design. Previous theoretical works^15–17^ have found that this procedure results in dielectric bowtie cavities (DBCs) but inverse design is prone to yield unrealistic designs unless constrained by the limitations of materials and fabrication, which was pointed out in recent theoretical works^17,18^. Here, we first measure the fabrication constraints of a state-of-the-art nanofabrication process and then include these constraints directly in the topology optimization to design a compact nanocavity. This interweaving between design and fabrication results in the novel and, importantly, realistic cavity shown in Fig. 1a, b, which we fabricate with high fidelity (Fig. 1c). While nanotechnology based on lithography is particularly suited to accurately map designs onto fabricated devices, realizing fabrication-constrained topology-optimized devices is an outstanding challenge in inverse design that was not attempted in any field of research or engineering until now.

The underlying principle of DBCs is local field enhancements due to the electromagnetic boundary conditions across material interfaces^15–17,19–24^. They demand that the tangential component of the electric field, **E**, and the normal component of the displacement field, **D** = *ϵ***E**, are continuous. This implies that a semiconductor bridge surrounded by void features, cf. Fig. 1a, b, can confine light inside the material, which is crucial to enhance the interaction with embedded emitters^1^ or material nonlinearities^23^. Besides the fundamentally different confinement mechanism, DBCs differ from previous cavity paradigms in several ways. First, the small mode volume of nanometer-scale DBCs implies strong light-matter interaction without resorting to extremely high quality factors, *Q*, thus enabling applications requiring wide bandwidths such as nanoscale light-emitting diodes, few-photon nonlinearities with short pulses^5,7,23^, quantum optics with broadband emitters^25^, and optical interconnects^26^. Second, the field enhancement of DBCs relies on the proximity to material boundaries, which implies that their modes are very sensitive to the precise size and shape of the bowtie^17,22–24,27,28^. Smaller bridges reduce *V*, immediately implying that a new frontier of nanocavity research is concerned with reducing the smallest feature size allowed by the nanofabrication process. This is in contrast to previous work that aimed to increase *Q*, since *V* was believed bounded at the diffraction limit in dielectrics^10,29^, which in turn required reducing structural disorder rather than the critical dimension^30,31^. Third, the presence of material discontinuities in few-nanometer devices makes the numerical modeling of bowtie cavities very challenging, requiring a very small mesh size^23^. Even the smallest discontinuity in the outline of the geometry implies a mode volume of zero^24^. Such numerical artefacts arise from the well-known electric-field divergences at sharp tips and corners^5,9,23,32,33^. This also implies that the commonly used definition of the mode volume, which normalizes to the maximum, is not generally applicable to DBCs because it gauges unintended lightning-rod surface effects rather than the effect of confining light inside the material^24^.

Experimental demonstrations of dielectric confinement of light below the diffraction limit have been reported before^34^ but the claimed mode volumes in these preliminary experiments were later shown^24^ to be underestimated by at least one order of magnitude due to numerical errors and inconsistencies. Another issue with previous experiments is that they employed a V-groove whose tip enhances the optical intensity by a lightning-rod surface effect that falls off exponentially inside the dielectric and does not provide subdiffraction confinement away from the surface. The lightning-rod confinement at surfaces is not useful for semiconductor devices relying on an increased light-matter interaction inside the material and does not signify a globally confined mode but only a local perturbation. Indeed, the measurements in previous work found modes much larger than the diffraction limit, which is directly observed in the measurements of the near-field 15 nm above the surface^34^, where any lightning-rod spikes, even if present, would have decayed below measurable levels. In summary, confinement of light deep below the diffraction limit was not demonstrated in previous experiments. Furthermore, it is important to realize that since the field diverges at sharp tips and corners, the commonly used definition of the volume that normalizes to the field maximum is not robust. For example, this max-evaluation of the mode volume predicts that the mode volume of a dielectric cube goes to zero because of field divergences at the corners, regardless of the size of the cube, and without affecting the field except exactly at the corners. This means that lightning-rod surface effects are particularly sensitive to numerical artefacts and we therefore evaluate and report the mode volume in the center of our silicon bridges as devised in ref. 24. This quantity is robust against surface effects such as non-vertical sidewalls as shown explicitly in Supplementary Section 1, and it is the relevant quantity describing enhanced spontaneous emission from embedded emitters such as defect centers^35^. Here, we use fabrication-constrained topology optimization to design a compact silicon nanocavity with a mode volume 12 times smaller than the diffraction limit evaluated in the geometric center of the cavity. We use scanning electron microscopy (SEM) and far-field spectroscopy to obtain a consistent picture of theory and experiment for both structural and spectral properties. Finally, we use near-field spectroscopy to establish an upper bound to the experimentally realized mode volume well below the diffraction limit. Our work therefore constitutes the first experimental demonstration of confinement of light below the diffraction limit in dielectric cavities.

## Results

### Inverse design and nanofabrication

We use carefully measured fabrication constraints as input to size- and tolerance-constrained topology optimization^17,18^ aiming to maximize the projected local density of optical states^1^ (LDOS) at the geometric center of the domain, which is forced to be solid. The procedure for measuring the fabrication constraints is detailed in Supplementary Section 2. This ensures that the optimization is protected from local lightning-rod effects at the surface and instead achieves robust confinement inside the dielectric bowtie^24^. Our devices are based on 240 nm crystalline (100) silicon membranes (*n* = 3.48) suspended in air, patterned with electron-beam lithography, dry etching, and selective vapor-phase hydrofluoric acid etching. We optimize a cyclic dry-etching process^36^ to minimize the critical dimension while tolerating periodic sidewall roughness in the form of scallops^37^, see Methods. We note that surface roughness and the size of the scallops could be reduced by hard etching masks. The fabrication constraints are quantified as a set of critical dimensions, which we define through minimum attainable radii. For our process, we find the radius of curvature of any solid feature, *r*_*s*_ ≥ 10 nm, and any void feature, *r*_*v*_ ≥ 22 nm. The critical radii are limited by proximity effects during electron-beam lithography but it is possible to go below these limits with manual shape modifications of the exposure mask, see Supplementary Section 3. From systematic tests we find that it is possible to obtain a mean bowtie bridge width of 8 nm in a localized area, which we include as a third critical radius of curvature, *r*_*c*_ ≥ 4 nm, at the center of the design domain. The topology optimization targets a maximum LDOS around *λ* = 1550 nm by tailoring the material layout in a small square domain with 2*λ* side length. We fabricate DBCs based on these parameters and the resulting structures show excellent agreement with the designed geometry as displayed in Fig. 1c. The high fidelity of the fabricated structures compared to the design demonstrates explicitly the value of directly including the measured fabrication constraints in the topology optimization.

The quasi-normal mode of the structure (including the tethers used to suspend the cavity, cf. Fig. 1c) is calculated using a finite-element method and we project the electric field ∣**E**∣ on the symmetry planes of the structure in Fig. 1a, b. We calculate the effective mode volume^38^

1 $$\frac{1}{V}={{{{{{{\rm{Re}}}}}}}}\,\left[\frac{{\epsilon }_{r}({{{{{{{{\bf{r}}}}}}}}}_{0}){{{{{{{\bf{E}}}}}}}}({{{{{{{{\bf{r}}}}}}}}}_{0})\cdot {{{{{{{\bf{E}}}}}}}}({{{{{{{{\bf{r}}}}}}}}}_{0})}{\int _{V}{\epsilon }_{r}({{{{{{{\bf{r}}}}}}}}){{{{{{{\bf{E}}}}}}}}({{{{{{{\bf{r}}}}}}}})\cdot {{{{{{{\bf{E}}}}}}}}({{{{{{{\bf{r}}}}}}}})\,dV+i\frac{c\sqrt{{\epsilon }_{r}}}{2\omega }\int _{S}{{{{{{{\bf{E}}}}}}}}({{{{{{{\bf{r}}}}}}}})\cdot {{{{{{{\bf{E}}}}}}}}({{{{{{{\bf{r}}}}}}}})\,dA} \right],$$

with **E**(**r**) and *ϵ*_*r*_(**r**) the electric field and dielectric constant at position **r**, respectively. *ω* is the complex angular eigenfrequency of the cavity mode and *c* is the speed of light. The mode volume is in general a function of position, but for this to be a robust and useful definition, we evaluate it at the center of the cavity, **r**_0_. We find *V* ~ 0.08(*λ*/(2*n*))^3^ and *Q* ~ 1100, around *λ* = 1551 nm. The volume integral is over the entire simulation domain, while the surface integral should be evaluated on the outer boundaries and in practical calculations only constitutes a minor correction^38^ for cavities with *Q* ≫ 10, such as our DBCs.

We stress that the bowtie, along with all other details, are emergent features arising entirely from the inverse design process. Similarly, the fact that the mode volume falls deep below the diffraction limit of *V* = (*λ*/(2*n*))^3^ is a result of our algorithm aiming to optimize the LDOS in a limited domain. Gondarenko et al.^15^ used inverse design to obtain the first DBC with confinement in air, and concluded that the bowtie shape reduces *V* as well as that the ring gratings increases *Q*. While these features can be identified qualitatively from our inversely designed cavities, the performance of intuition-based cavity designs is inferior to topology-optimized structures^17^. Although the very large parameter space for the inverse-design algorithm makes it impossible to ascertain if the resulting design is a global optimum, it is interesting to note that the angle of the bowties are ~90°, that the bridge width equals 2*r*_*c*_, and that the voids surrounding the bridge are rounded with ~*r*_*v*_. These are exactly the parameters that were recently established as the global optimum for confinement of light inside bowties^24^ and the minor deviations reflect the fact that our algorithm optimizes LDOS, i.e., it targets not only the smallest *V* but, at the same time, the largest *Q* for the given footprint and our fabrication constraints.

Although we unambiguously demonstrate photon confinement deep below the diffraction limit, the modes are so compact that we cannot measure the precise size of the mode^39^. Therefore, measuring the width of the fabricated silicon bridge is crucial for rigorously comparing theory and experiment for DBCs. However, the bridge width of a few nanometers is close to the resolution limit of conventional microscopy methods, such as scanning electron microscopy. We therefore fabricate three sets of DBCs, each of which subject to a global geometry-tuning, *δ*, of the entire mask, thereby shrinking the exposed areas (air) in incremental steps of 2 nm as shown in Fig. 1d. In order to further validate the yield and reproducibility, we fabricate and characterize six nominally identical copies of each geometry-tuned device. Representative SEM images of each of the three geometry-tuned devices are shown in Fig. 1e–g and the 2 nm systematic variations are clearly observed in the change of the fabricated bowtie dimensions. We measure a mean bowtie bridge width of 8 nm, 10 nm, and 16 nm, for the three geometry-tuned devices, respectively. See Methods and Supplementary Section 4 for further details on the SEM characterization, and Supplementary Section 6 for an overview of devices characterized in this work.

### Far- and near-field measurements

**Fig. 2:**
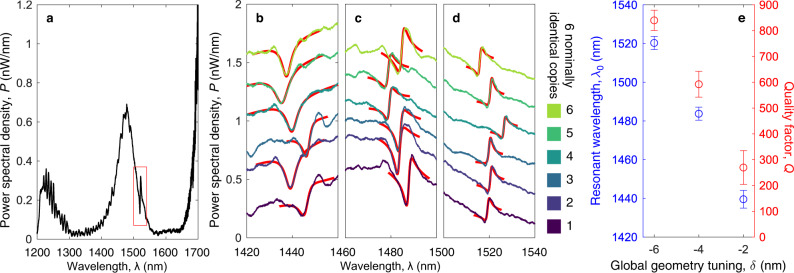
Optical far-field characterization of dielectric bowtie cavities. **a** Broadband spectrum of a cavity with *δ* = − 6 nm. The cavity mode interferes with a background mode yielding a Fano resonance centered at *λ* ~ 1520 nm, highlighted by the red box. **b**–**d** Spectra for six nominally identical devices for each tuning *δ* = { − 2, − 4, − 6} nm. The spectrum of each copy is offset incrementally by 0.25 nW nm^−1^ for clarity. The full spectrum for cavity 5 is shown in **a**. The red lines show fits to the Fano lineshape. **e** Mean and standard deviation of resonant wavelength *λ*_0_ (blue, left) and quality factor *Q* (red, right) against *δ*, extracted from the fits in **b**–**d**.

We characterize the devices using confocal cross-polarized microscopy (see Methods) and a representative reflection spectrum is shown in Fig. 2a. This spectrum shows the cavity mode as a feature around 1520 nm. The DBC mode interferes with the low-*Q* vertical cavity mode formed by the (~3 μm) air gap between the silicon device layer and the silicon substrate. This results in a Fano resonance, which is well known from confocal characterization of nanocavities^40^. The Fano line shape takes the form

2 $$F(\omega )={A}_{0}(\omega )+{F}_{0}\,\frac{{\left[q+2(\omega -{\omega }_{0})/{{\Gamma }}\right]}^{2}}{1+{\left[2(\omega -{\omega }_{0})/{{\Gamma }}\right]}^{2}},$$

where *ω* is the frequency, *ω*_0_ is the DBC resonant frequency, Γ is the linewidth, *A*_0_(*ω*) is a linear function representing the background low-*Q* mode, *q* measures the relative amplitudes between the main and the background modes, and *F*_0_ is a constant scaling-factor. The spectra for all six copies of each of the three geometry-tuned devices shown in Fig. 1e to g are displayed in Fig. 2b–d. We fit the Fano model locally around each resonance and extract *ω*_0_ and the quality factor *Q* = *ω*_0_/Γ for all 18 devices of the three global geometry-tuning parameters. Figure 2e shows the mean and standard deviation of the resonant wavelength, *λ*_0_, and *Q*, for each *δ*. We obtain a mean spectral shift Δ*λ* = (40.4 ± 0.6) nm between each incremental value of *δ* = − 2 nm from a linear fit and find that the standard deviation of the resonance shift of each set of geometry-tuned devices is ≤ (4 ± 0.6) nm. That is, the six nominally identical copies has spectral shifts ≤ Δ*λ*/10, which corresponds to the devices being identical within ∣*δ*∣ ≤ 0.2 nm.

**Fig. 3:**
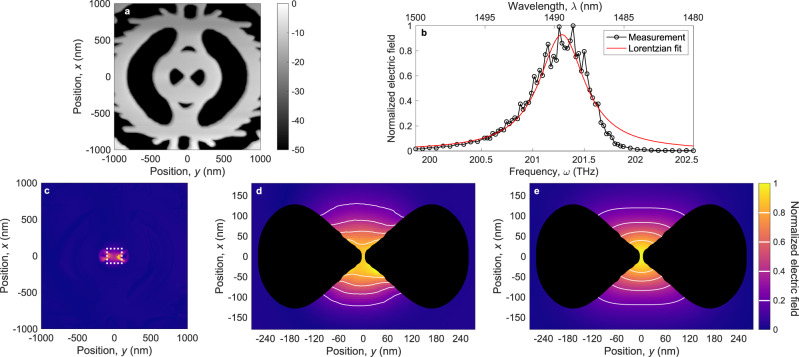
Scattering-type scanning near-field optical microscopy (s-SNOM) of cavity mode. **a** Topography measured by atomic force microscopy (AFM). **b** Spectrum measured of amplitude of scattered field with Lorentzian fit, *λ*_0_ = (1489.4 ± 0.1) nm and *Q* = 370 ± 40. **c** s-SNOM signal on resonance demonstrating strong field localization with excellent suppression of background noise. The white dotted box highlights a square domain with its side length given by the diffraction limit, *λ*_0_/(2*n*_Si_) ~ 200 nm. The strong signal in the cavity voids arises due to complex interactions between the AFM probe and the cavity mode. **d** Normalized measured amplitude of the light scattered from the cavity surface with the voids blacked out. **e** Numerical simulation of experiment, $f$(*σ* = 37 nm) ∗∣**E**_*c*_∣^2^, confirming photon localization below the instrument response function as explained in the main text. The s-SNOM measurements presented here were performed on cavity copy 3 (*δ* = − 4 nm), which has a mean bowtie width of 10 nm.

While far-field measurements give important insights into the spectral properties of DBCs, they do not allow extracting information about the mode shape and confinement. We therefore interrogate the near-field immediately above the DBCs using a scattering-type scanning near-field optical microscopy (s-SNOM), where a continuous-wave laser is focused on an oscillating atomic force microscope (AFM) silicon tip scanning across the DBC. Figure 3a shows the measured topography, which provides a clear image of the device but also shows that the tip penetrates into the void features, implying that the measured geometry is convolved with the function describing the tip. For the near-field optical characterization we use a pseudo-heterodyne interferometric detection scheme, which strongly suppresses interference with the far-field background^41^. This experiment allows recording the optical spectrum of the cavity mode without exciting the low-*Q* background resonance. Figure 3b shows the measured amplitude at an effective height of 5 nm above the surface at the center of the DBC. We model the measured cavity mode using a Green-tensor formalism treating the tip as a polarizable sphere and find that the measured amplitude is modulated by the intensity of the cavity mode^42^. From a Lorentzian fit in the frequency domain we obtain *λ*_0_ = (1489.4 ± 0.1) nm and *Q* = 370 ± 40. The reduction in *Q* arises since the s-SNOM tip acts as an additional loss channel for the cavity so the s-SNOM experiment measures a loaded *Q*. The deviation from a Lorentzian lineshape may be due to nonlinear interactions or coupling with the near-field tip^43^.

When continuously exciting the DBC with a laser tuned to the cavity resonance while scanning the position, we can map out the spatial structure of the cavity mode. The result, which is shown in Fig. 3c, shows that the mode is strongly localized at a single hotspot. The near-field measurements in Fig. 3c show enhanced fields at the edges of the void features on the sides of the silicon bridge. These scattering fields arise because the tip goes down into the holes and therefore scatters not only surface fields but a complex combination of the surface field and the field in the voids, see Supplementary Section 5. We disregard the data obtained when the tip falls into the voids in the following analysis to facilitate a direct comparison between the measured field above the device to theoretical predictions. Figure 3d shows a high-resolution map of the measured normalized scattered field amplitude with the regions above the voids blacked out. The measured field cannot be compared directly to the calculated quasi-normal mode shown in Fig. 1a, b because the measured amplitude probes the intensity of the cavity mode and, in addition, because of the influence of the tip. We model the tip instrument function, *f*(*σ*), as a Gaussian of standard deviation *σ* and maximize the overlap between measured and calculated field through the Bhattacharyya coefficient, $t=\sum \sqrt{|{{{{{{{{\bf{E}}}}}}}}}_{s}|\,\cdot (|{{{{{{{{\bf{E}}}}}}}}}_{c}{|}^{2} * f(\sigma ))}$, where ∗ denotes convolution and **E**_*s*_ (**E**_*c*_) is the measured (calculated) field 15 nm above the surface. The tip has a nominal radius of curvature of 10 nm and probes the field when the edge is 5 nm above the surface. Both fields are normalized, $\sum|{{{\bf{E}}}}_{s}|=\sum|{{{\bf{E}}}}_{c}|^{2} * f(\sigma )=1$, with the sum being over all pixels. This analysis yields *σ* = (37 ± 5) nm and *t* = 0.984, and Fig. 3e shows the convolution of the corresponding instrument function, *f* (*σ* = 37 nm), with the calculated field amplitude 5 nm above the structure. The large overlap indicates an excellent agreement between theory and experiment. The instrument function has a full width at half maximum of $2\sqrt{2\log (2)}\sigma$ = 87 nm, which is broader than the DBC mode size, so the measurement gives an upper bound to the mode volume, which falls below the diffraction limit, *V* = (*λ*/(2*n*))^3^. This corresponds to a cube with a side length of ~200 nm as indicated by the dotted white box in Fig. 3c. Notably, even after the expansion of the mode above the cavity and after broadening by the instrument function of the near-field tip, the raw data shows an optical mode confined well below the diffraction limit. Additional s-SNOM measurements (see Supplementary Section 5) on a device of different global geometry-tuning, *δ* = − 6 nm (corresponding to a mean bowtie width of 16 nm), also yields the largest overlap, *t* = 0.991, for the same instrument function *σ* = 37 nm. The overlap between the two measurements is *t* = 0.996, which further confirms that the DBC mode is localized below the instrument function. These results constitute the first direct experimental measurement of subdiffraction confinement of light in a dielectric structure.

## Discussion

Strongly confining light inside dielectrics, as opposed to in air, vacuum, or at material boundaries, is central to applications relying on enhancing the light-matter interaction. Our work demonstrates for the first time the advantages of including measured fabrication constraints in topology optimization. This sets a new standard for photonic nanotechnology in the quest for globally optimal structures^28,44^ and demonstrates for the first time photon confinement inside dielectrics below the diffraction limit without intrinsic limits on *Q*. The directly optimized LDOS of our cavity corresponds to an enhancement of the light-matter interaction by a Purcell factor^5^ of 6 × 10^3^ over a bandwidth of up to 2 nm. This large bandwidth is needed for nonlinear optics and optical interconnects^26^ and appears on purpose in our design due to the compact device footprint^16,17^ of 4*λ*^2^. The combination of our cavities with embedded emitters may enable direct studies of very large and broadband Purcell factors. Many commonly studied quantum emitters are unsuitable due to size constraints, e.g., self-assembled quantum dots^1^ are generally larger than the bowtie bridges demonstrated in our work, but our devices are directly compatible with narrow-linewidth erbium dopants in silicon^45^. Extending the design domain would result in much higher *Q*, and our work therefore not only demonstrates unprecedented levels of photon confinement inside dielectrics, it also paves the way for experiments in extreme regimes of light-matter interaction, which in turn can suppress quantum decoherence due to phonons^46^.

We note that the semiconductor technology nodes, such as the current “5-nm node”, of the semiconductor industry no longer describe the smallest features in integrated circuits defined by lithography^47^. In fact, the current industry roadmap for lithography does not aim to go below 8 nm before 2034. The ability to fabricate highly optimized devices with 8 nm dimensions and high aspect ratios is therefore unlocking new experimental regimes throughout most areas of semiconductor nanotechnology^48^, including nanophotonics^2^, cavity optomechanics^6^, nanoelectromechanics^49^, and quantum photonics^1^.

## Methods

### The inverse design process

For the inverse design procedure we model the physics using Maxwell’s equations in a finite volume of space, assuming time-harmonic field behavior. We exploit the three-fold spatial symmetry of the DBC structure to reduce the model size and truncate the modeling domain using symmetry conditions and first-order absorbing boundary conditions^17^. The model is discretized and solved using the finite-element method with first-order Nedelec elements^50^. The problem of designing a DBC is solved using topology optimization by recasting it as a continuous constrained optimization problem^51^. In this process we select a subset of the model domain, i.e., the design domain, and introduce one spatially constant continuous design variable per finite element in the design domain. We apply a filtering and thresholding procedure^18,52^ to regularize the design. The filtered and thresholded design variables are linked to the model through a material interpolation scheme^53^. Hereby, the design variables control the material distribution. The optimization problem is solved using the globally convergent method of moving asymptotes^54^. For the domain considered in this work, we choose a fixed membrane thickness of 240 nm and restrict the design to only vary in the (*x*, *y*)-plane by linking the design variables along the *z*-direction^12,55^. Before the design process is executed, we specify the design domain, the measured minimum radii of curvature of the solid and void phases in the design as well as at the center, and further specify the targeted cavity-resonance wavelength and the position of the mode extremum in the cavity. Otherwise, we allow the design to emerge freely from the design process.

### Fabrication processes

A 25-by-25 mm chip is cleaved from a silicon-on-insulator wafer with a 240 nm (100) device layer and a 3 μm buried oxide. It is cleaned sequentially with de-ionized water, acetone, isopropanol (IPA), and dried with dry N_2_. The sample is dehydrated for 5 min at 200 °C and ~65 nm chemically semi-amplified resist (CSAR) is spin-coated from CSAR6200.04 (CSAR6200.09 diluted 1:1 in anisole) at 6000 rpm for 60 s followed by a 5 min softbake at 200 °C. Six nominally identical copies of the cavity layout (56 combinations of local mask corrections and global geometry-tuning) are exposed uniformly on a 100 keV 100 MHz JEOL-9500FSZ electron-beam writer with current *I* = 202 pA, dose density *D*_0_ = 3 aC nm^−2^, and shot pitch, *p* = 1 nm. The samples are developed for 60 s in AR-600-546 (amyl acetate), cleaned in IPA, and dried with dry N_2_ in an automatic Laurell EDC 650 puddle developer for high reproducibility. All devices are separated by 25 μm to reduce proximity effects.

The patterns are transferred to the device layer with 10 cycles of a modified version of the CORE-sequence^36^ operated at +20 °C. This process is a low-power switched reactive ion etching process using SF_6_ for the etch and oxygen for sidewall passivation, thus avoiding fluorocarbon residues. Specifically, we fine-tune the process to achieve an aspect ratio of 30 from the thin softmask required by lithography. To improve the mask selectivity we reduce the platen power of the R-step from 10 W → 8 W and to reduce sidewall erosion we increase the O-step (passivation) from 3 s → 4 s. Lastly, we reduce the SF_6_ flow in the E-step from 15 sccm → 10 sccm and modify the duration of this step from 73 s → 72 s. The resist is removed with 1165 Remover (N-Methyl-2-pyrrolidone) followed by IPA and dried with dry N_2_. The sample is then cleaned for 10 min in a Tepla 300 barrel asher with 400 sccm O_2_-flow and 70 sccm N_2_-flow at 1 kW reaching a maximum temperature of 72 °C. The buried oxide is etched in anhydrous hydrofluoric-acid (99.995 %) vapor using ethanol as catalyst at a process pressure 131 Torr in an SPTS Primaxx uEtch enabling both pressure and temperature control throughout the release. The sample is baked for 5 min at 200 °C prior to the release etch to avoid residues.

### Scanning electron microscope characterization

We measure the dimensions of the fabricated structures by comparing a combination of top-view and tilted SEM images analyzed with detailed image analysis, presented and discussed in Supplementary Section 4. We measure the width of the bowties as 13 nm, 15 nm, and 21 nm from the top-view SEM images of the three sets of devices presented in Fig. 1e–g, respectively. Furthermore, we use multiple tilted views to estimate the width at the bottom of the bowties, which we find is ~10 nm narrower than at the top. This implies a negative sidewall angle ~1° of all devices and a mean width of the bowtie bridges of 8 nm, 10 nm, and 16 nm, for the three geometry-tuned devices, respectively, consistent with the critical radius of curvature imposed on the topology optimization. Supplementary Section 1 presents careful numerical simulations of the fabricated dimensions, which both includes the sidewall angle as well as variations of the dimensions of the calculated structure. This confirms that the mode volume in the center of our tolerance-constrained DBC-design remains robust to variations and is deep below the diffraction limit.

### Confocal cross-polarized microscopy setup

A supercontinuum laser (NKT Photonics SuperK Compact) is focused on the cavity through a NA = 0.4 microscope objective. The scattered light is collected through the same objective and measured with an optical spectrum analyzer (AQ6370D Yokogawa), wavelength range *λ* = [1200, 1700] nm. The excitation polarization is controlled with a *λ*/2-plate and light is collected through a linear polarizer rotated 90° to reduce specular reflections. Both excitation and collection is rotated 45° to the main optical axis of the cavity (along *x* in Fig. 1a).

### Near-field optical measurements

We use an s-SNOM (Neaspec, neaSNOM), equipped with a pseudo-heterodyne module, in reflection mode to map the DBC modes in the near-field. The incident light from a tunable continuous-wave laser (Santec, TSL-710) is focused on a silicon AFM probe (NanoWorld, Arrow-NC) with a nominal tip radius of 10 nm. The probe is used in intermittent contact mode at a frequency *f*_0_ = 280 kHz oscillating with an amplitude of 60 nm. The amplitude of the scattered signal depends nonlinearly on the height above the sample due to the near-field contribution, therefore, demodulating at 4*f*_0_ with a lock-in amplifier yields the near-field signal at the smallest height (~5 nm) above the surface while strongly suppressing contributions from the far-field background. The laser is s-polarized, which is aligned along the *x*-axis of the DBC (see Fig. 1a), to minimize the perturbation from the tip and to excite the cavity most efficiently. A polarizer is placed in front of a photoreceiver (New Focus, 2053-FS) to select the s-polarization of the scattered field. We determine the resonant wavelength in the near-field from a Lorentzian fit to the near-field spectrum obtained from a fixed position in several spatial maps obtained around the bowtie for a number of wavelengths in a 20 nm band, see Supplementary Section 5 for further details.

## Supplementary information

Supplementary Information

## Data Availability

Data is available upon request.
