# Supplementary material for: Nanometer-scale photon confinement in topology-optimized dielectric cavities
Source: Nat Commun. 2022 Oct 21;13:6281. doi: 10.1038/s41467-022-33874-w (PMC9587274; doi:10.1038/s41467-022-33874-w)
Supplement: Supplementary file 1 — Supplementary Information [file 41467_2022_33874_MOESM1_ESM.pdf]

Supplementary information for
Nanometer-scale photon confinement in topology-optimized dielectric cavities

1 Numerical simulation of dielectric bowtie cavities

The mode volume is defined as the inverse of the normalized energy density evaluated at a position $\mathbf{r}_0$. This follows, e.g., from the Purcell factor describing the enhancement of the decay rate in a nanostructure compared to a homogeneous medium, i.e., the ratio of the local density of optical states (LDOS) to the density of optical states (DOS) [1–3]. For nanocavities with high Q and/or small V it is often an excellent approximation to assume that the LDOS is dominated by a single cavity mode, thus neglecting continuum modes. The LDOS is a function of frequency, polarization, and position and these dependencies remain crucial in the single-mode approximation. The mode is a quasi-normal mode and can be calculated in several ways, for example by exciting the cavity with a dipole at position $\mathbf{r}_0$ and computing the LDOS directly [4, 5], which is the method we employ in our topology optimization. The quasi-normal mode can also be calculated from the eigenfrequency, which is what we employ everywhere else in this work. The eigenfrequency is a linear solution, which can be scaled arbitrarily and therefore it must be normalized to yield an absolute energy density. This can be done in several ways [6, 7], and in practical calculations it is convenient to use Eq. (1) in the main text.

We use finite-element modelling [8] to simulate our structures. Figure S1 shows the numerical model solved in a finite-element model with COMSOL Multiphysics 5.6. Only 1/8th of the dielectric bowtie cavity (DBC) is simulated due to symmetries, except for our simulations of a negative sidewall angle, which breaks the out-of-plane symmetry enabling only two symmetry planes. The structure is meshed using a different mesh size around the central bowtie and the mesh size in all regions is determined by convergence tests, which results in a finer mesh around the bowtie.

Figure S2 shows the results of the numerical simulations of the DBC design. Rather than the Manhattan design outline with all vertices at 90° to control the discretization of the electron-beam lithography, a smoothed outline is extracted from the topology optimization for the numerical calculations [5, 9]. Even if the polygons are rendered with high resolution, the discontinuities at vertices will introduce numerical artefacts as shown in Figs. S2**a**-**b** where, e.g., the spots highlighted by red circles contains dielectric wedges, which will diverge with finer mesh [10–16] resulting in an apparent enhancement of the mode volume locally, here by ~ 50 %. It is therefore not possible to solve this numerical problem with a finer mesh (Fig. S2**e**). Instead, higher-order polygon vertices must be used as shown in Figs. S2**c**-**d** to achieve a correct evaluation. In this case, the finite radius of curvature [9] imposed in the topology optimization directly limits the field amplitude at the interface. We have thus eliminated numerical artefacts from our model as well as in our design but we note that the evaluation of the mode volume at the geometric center of the cavity is in any case a robust quantity [17]. We show this explicitly by considering the effect of non-vertical sidewalls on the mode volume. Figure S3**a** and **b** compares the normalized electric energy, $\epsilon(r)|\mathbf{E}(r)|^2$, across the bowtie (the yz-plane), for vertical and non-vertical sidewalls, respectively. The energy is tightly confined

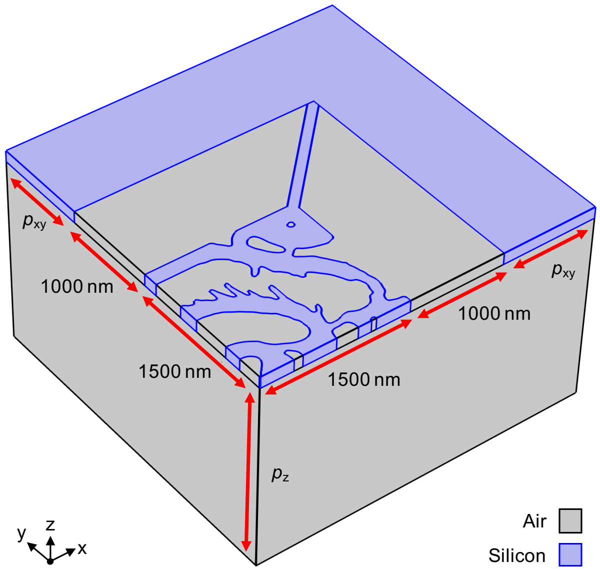

Fig. S1. Simulation domain for a DBC. The blue domains are silicon and the gray domains are air with the in-plane padding p_{xy} = 1.5 µm and the out-of-plane padding p_z = 3.1 µm. The xy-plane and xz-planes are perfect magnetic-conductor boundary conditions, and the yz-plane is a perfect electric-conductor boundary condition. For the angled-sidewall calculation, we do not employ the boundary condition on the xy-plane as this symmetry is broken. First-order absorbing boundary conditions are applied on the boundaries facing away from the cavity and are used for the surface integral when computing the mode volume as detailed in the main text.

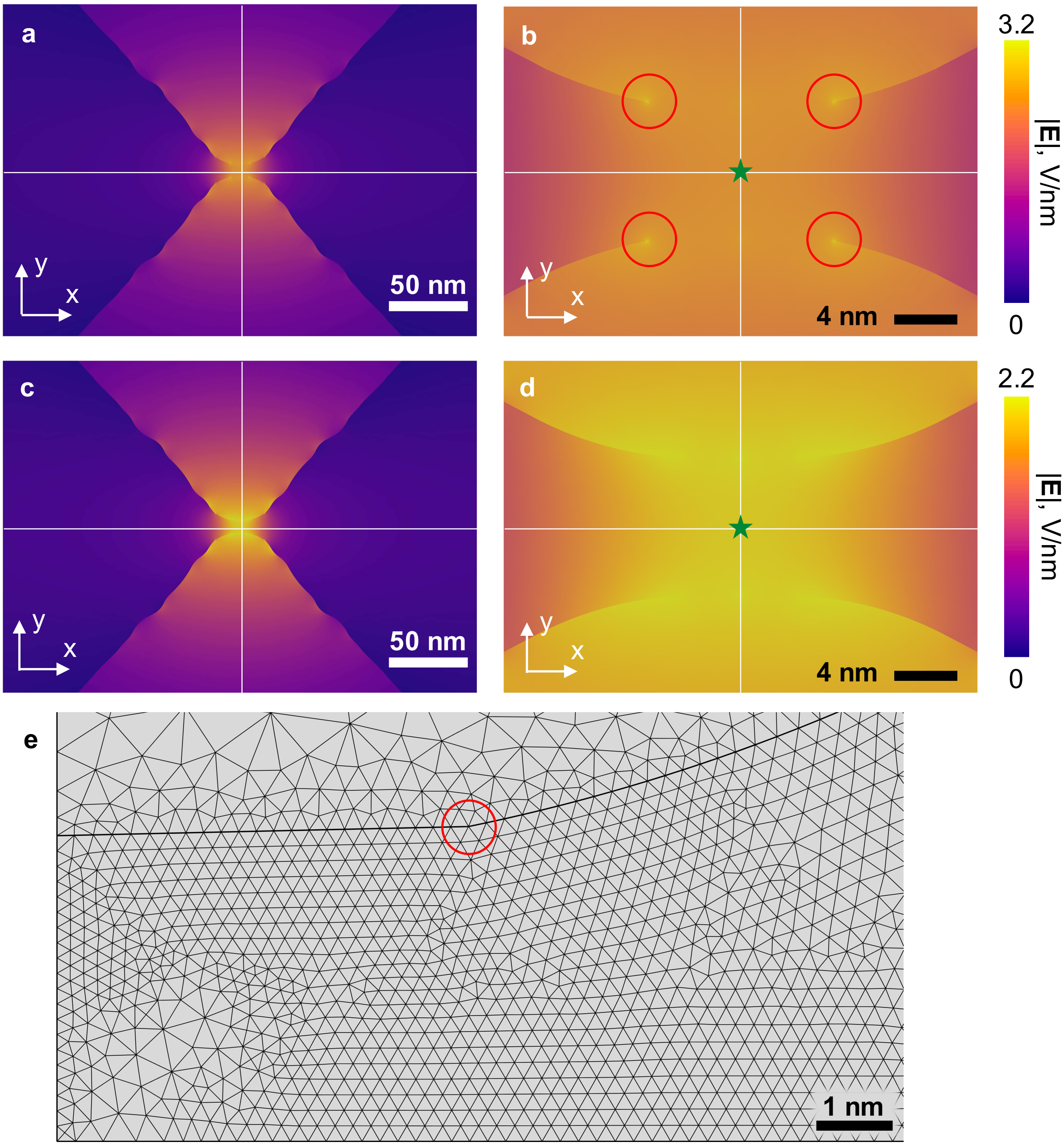

Fig. S2. Numerical challenges and local lightning-rod effects in DBCs. a, Numerical evaluation of the DBC where polygon vertices are not rounded to higher-order nodes in the geometry, and **b**, zoom-in around the bowtie with red circles indicating the lightning-rod enhancements of |**E**|. **c**, The same geometry evaluation with rounding of the problematic vertices. **d**, This results in a continuous field whose intensity at the center of the geometry is close to the maximum at the interface. The color scales are normalized by the peak field amplitude in **a**-**d**, and the field profiles are not smoothed. The white lines shows the symmetry planes through $x = 0$ and $y = 0$. **e**, The mesh of 1/4 of the structure around the bowtie with the problematic mesh elements highlighted (red circle).

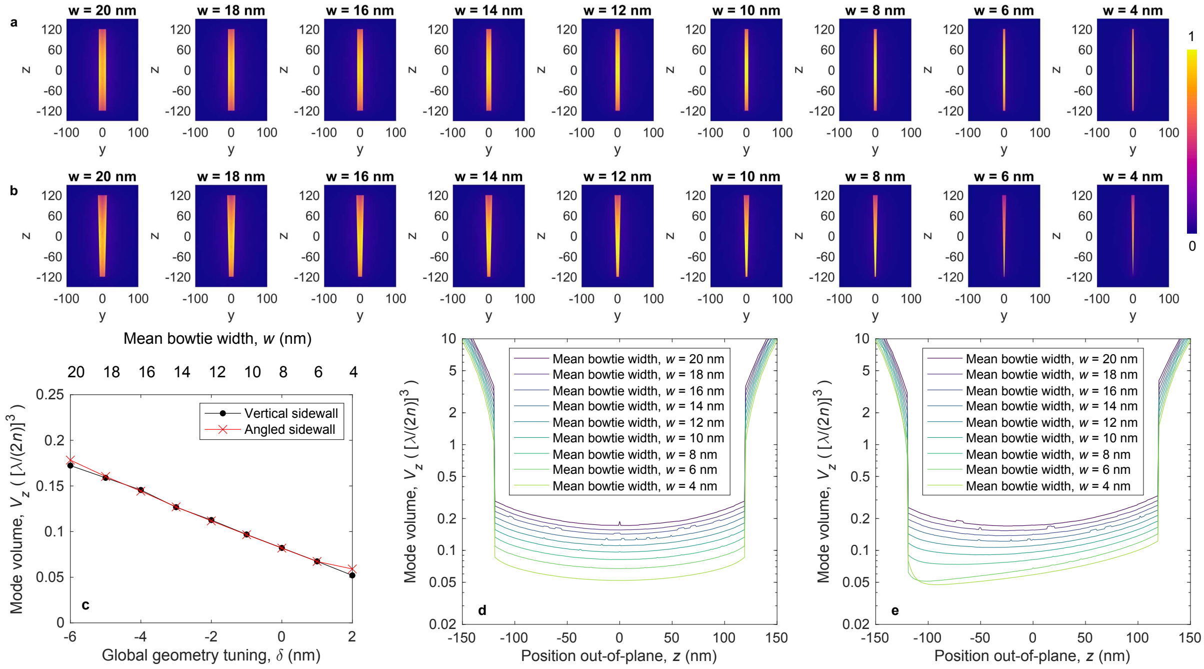

Fig. S3. Simulations of mode volume dependence on bowtie width and sidewall angle. a, Calculated electric energy $\epsilon(r)|\mathbf{E}(r)|^2$ in the yz-plane at $x = 0\,\mathrm{nm}$ as a function of bowtie width, w, assuming a vertical sidewall. **b**, Calculated electric energy in the yz-plane at $x = 0\,\mathrm{nm}$ as in **a** with a non-vertical sidewall where the width is 5 nm more narrow (wide) at the bottom (top). **c**, Effective mode volume according to Eq. (1) in the main text calculated for each of the 18 devices in **a** and **b** and mapped against global geometry-tuning, δ, and mean bowtie width. **d**-**e**, Effective mode volume evaluated for different positions along the z-axis for vertical (**d**), and non-vertical (**e**) sidewalls and the different bowtie widths shown in **a**-**b**.

and varies slowly within the dielectric. Note that the energy maximum occurs at the material boundary at $y \neq 0\,\mathrm{nm}$, consistent with the preceding discussions. Figure S3**c** shows the effective mode volume as a function of a global geometry-tuning, where all void features in the xy-plane are enlarged or shrunken, which in turn changes the mean bowtie width. Importantly, the mode volume is effectively independent of the sidewall angle when the mode volume is evaluated in the center, $\mathbf{r}_0$, which indicates that this definition is consistent and robust against surface effects.

The robustness of the definition of the mode volume evaluated at the cavity center, which we use in our work, is in contrast to evaluating the mode volume at the maximum of the field or in any other point in space. Figure S3**d** and **e** show V when evaluated at different out-of-plane (z) positions at $x = y = 0$ for vertical and non-vertical sidewalls, respectively. For a vertical sidewall, the smallest V is at $z = 0$. However, when the symmetry is broken by introducing a small deviation from verticality, the mode volume changes and smaller mode volumes seem to occur [18]. In addition, the mode volume would change significantly when moving the point of evaluation along y towards the material interfaces. This exemplifies some of the inconsistencies that emerge if an arbitrary point, even if it is the point of maximum field intensity, is used to evaluate the mode volume. The challenges and inconsistencies arising from evaluating the mode volume at the field maximum are particularly important for DBCs because they explicitly rely on field discontinuities at material boundaries but they can also emerge in conventional cavities [5, 16, 17, 19–26].

To summarize these discussions, the mode volume is a well-defined, robust, and consistent quantity when using Eq. (1) of the main text and evaluating the mode volume of a quasi-normal mode at the center of the cavity as detailed in refs. [2, 6]. We stress that this conclusion does not question the validity of the vast majority of previous works on conventional cavities because the field maxima typically occur in the center of such cavities so that the maximum evaluation leads to evaluation at the center. This conclusion does also not rule out the existence of surface effects [27], which can be very significant albeit hard to control experimentally and potentially governed by divergent fields that cannot be calculated numerically. It would then be crucial to evaluate the mode volume or the LDOS at the frequency, position, and polarization of the emitter for the particular experiment.

2 Determining the minimum radii of curvature of the nanofabrication

The radius of curvature (ROC) for void features, r_v, is evaluated with scanning electron microscopy (SEM) images on corners of fabricated triangles of different sizes as shown in Figs. S4**a** and **b**. In all cases, the ROC for void features is $r_v = 22$ nm. The ROC is to a good approximation independent of size and angle but it is easier to measure for larger features. Figure S4**c** shows a cross-section of millimeter-long lines etched with a clear-oxidize-remove-etch dry-etching sequence [28–30], demonstrating the high resolution of the fabrication process. The smallest resolved lines are bent due to electron exposure in the SEM. Figure S4**d** and **e** shows a DBC with the inset demonstrating that features of $r_s \geq 10$ nm can be realized away from the central bowtie. The bowtie is limited to $r_c \geq 4$ nm, which we achieve by carefully modifying the exposure mask locally.

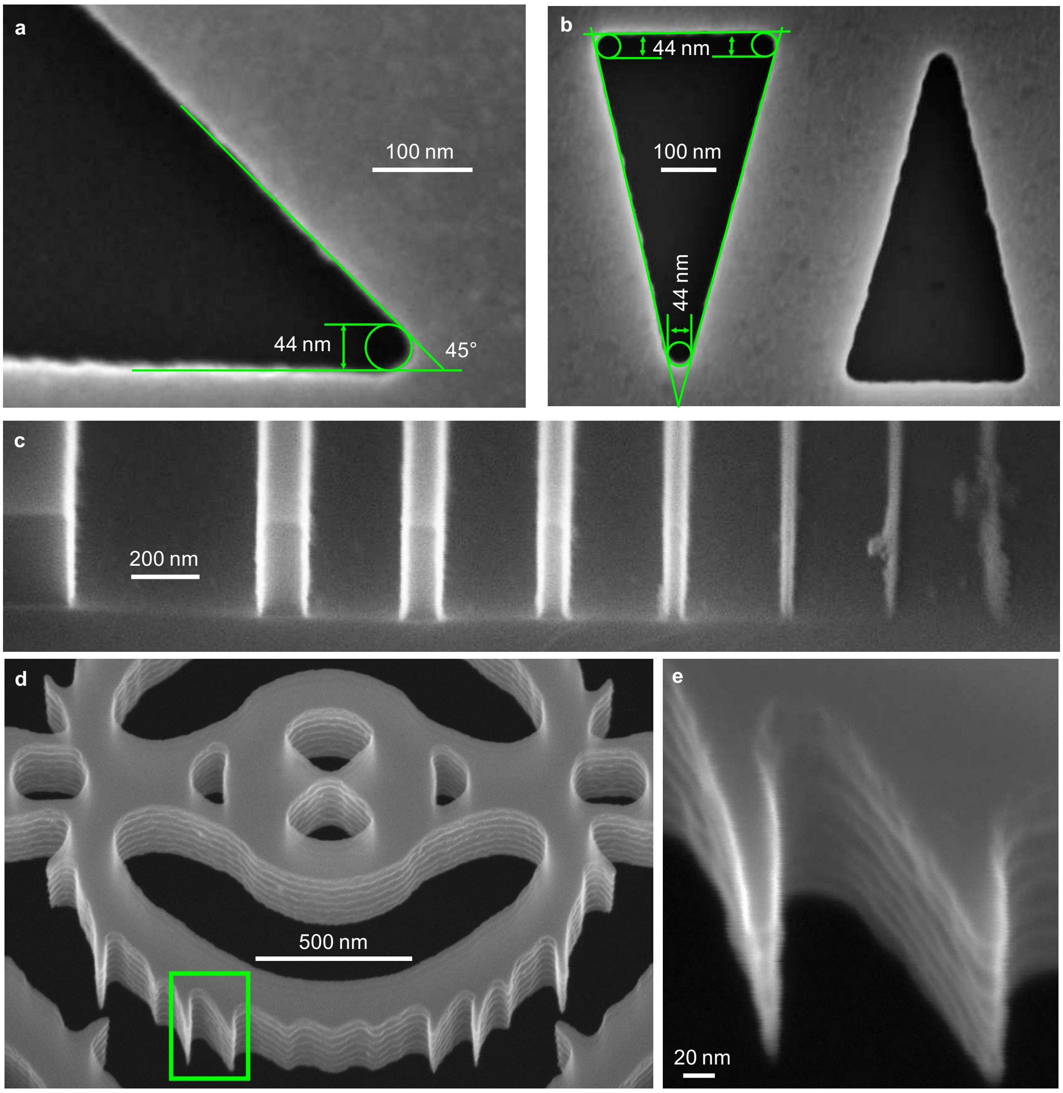

Fig. S4. Measurement of the radii of curvature (ROC) using scanning electron microscopy (SEM) images. a-b, ROC for void features measured to $r_v = 22$ nm on multiple vertices on several triangles of varying size and angles. **c**, Cross-section of 500 µm-long lines of varying width etched in silicon. **d**, Tilted SEM image of a fabricated DBC ($\delta = -6$ nm) where the sharp features in the void limited to $r_s \geq 10$ nm away from the bowtie are visible. The green rectangle highlights the region of the inset shown in **e**.

3 Tuning of exposure mask by global geometry-tuning and local mask corrections

We use a global geometry-tuning to systematically vary the cavity geometry and a local mask correction to fabricate devices with bowtie widths down to the limit set by the critical radius of curvature. To systematically characterize the effects of geometry-tuning and corroborate the observed spectral shifts for different geometry-tunings (Fig. 2 in the main text), we calculate the difference between the original designed mask and binarized top-view scanning electron microscopy (SEM) images of devices with different geometry-tuning, δ, as shown in Figs. S5**b-e**. Figure S5**a** shows the change of the exposure mask, where black pixels inside a given contour is exposed with electron-beam lithography. Figures S5**f-i** show tilted SEM images of the bowties. From Figs. S5**b-e** it can be seen that the outlines becomes less red and more blue with decreasing δ, indicating that the silicon features grow and the air features shrink as expected. We stress that while the top-view SEM images indicate that the bowties are successfully fabricated, the tilted SEMs in Figs. S5**f-i** clearly show that the bowtie in the untuned structure, $\delta = 0\,\text{nm}$, is not resolved. This means that both tilted and top-view images must be compared to evaluate the fabricated bowties, which is challenging when approaching the resolution limit of SEM.

Figure S6**a** shows the local mask corrections (LMC) applied to the lithographic exposure mask to achieve the narrow bowtie structure below the process resolution, ultimately limited by the critical radius of curvature $r_c \geq 4\,\text{nm}$. Figure S6**b-i** show top-view SEM images of the 8 local mask corrections before underetching of the structures. In this work we only analyze the device with LMC = 22 nm, since this yielded a fully etched bowtie for multiple global geometry-tunings. The global geometry-tuning was applied after the local mask correction.

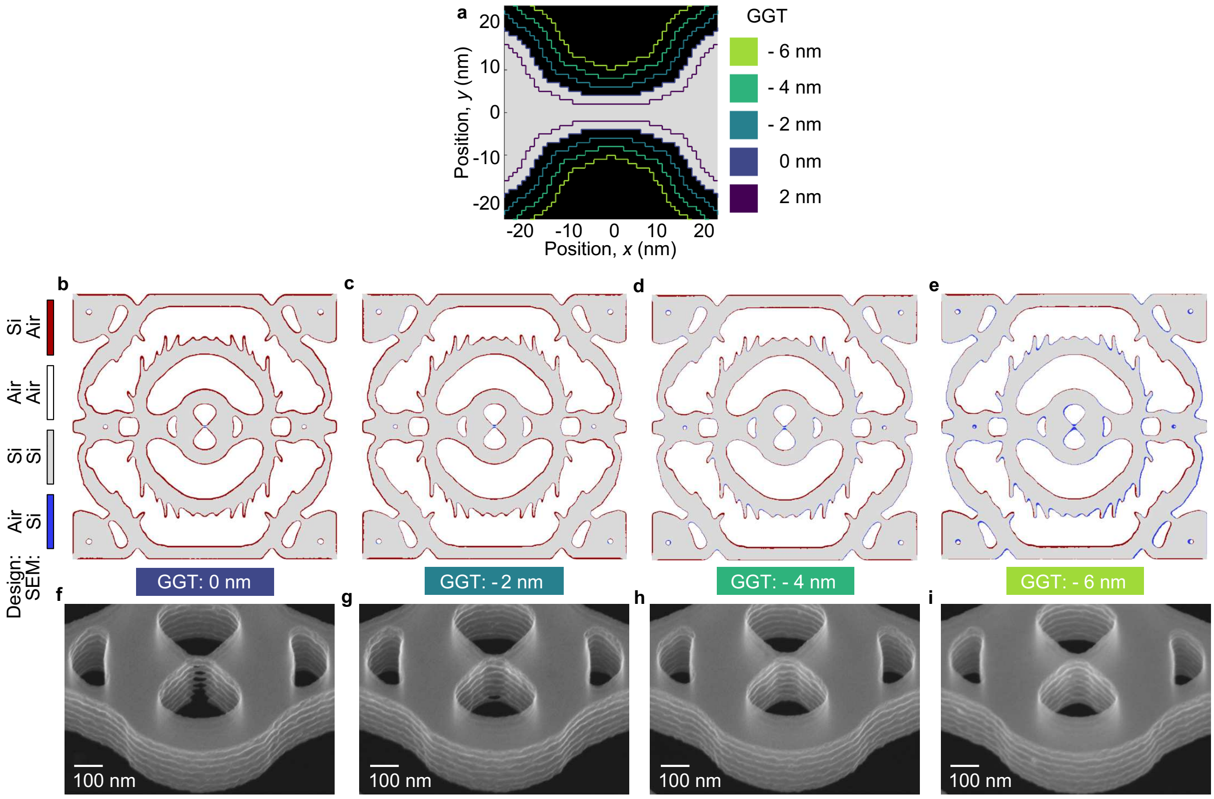

Fig. S5. Evaluation of fabricated DBC from image analysis of scanning electron microscopy (SEM) images. a, Extended global geometry-tuning, δ, from Fig. 1D. **b-e**, Difference between fabricated device and targeted mask obtained by applying a binary filter to a top-view SEM image of a device of each δ and subtracting the designed mask for each pixel, corresponding to the 40° tilted SEM images of the bowtie structures in **f-i**. **g-i**, are reproduced from Fig. 1 of the main text for clarity.

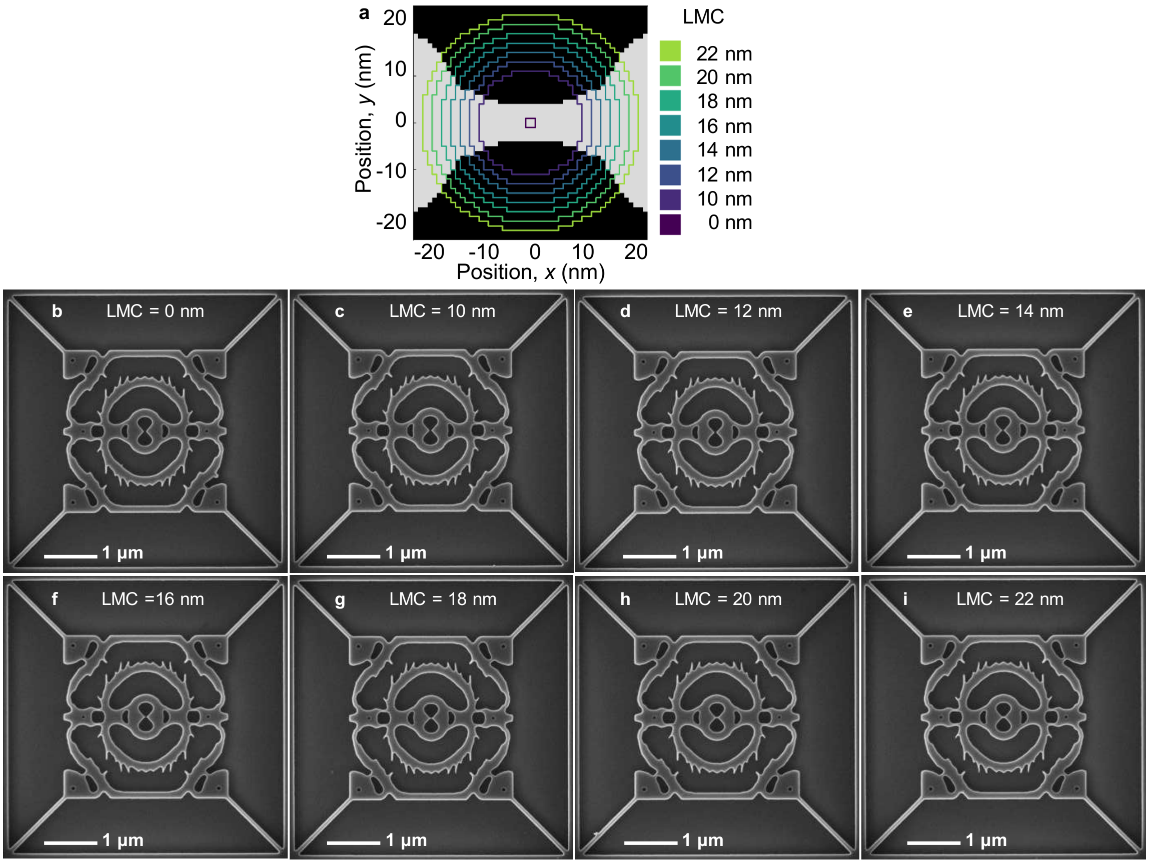

Fig. S6. Local mask corrections (LMC) around the bowtie. a, The outline of the structure (grey) contains features below the radius of curvature but the resolution of critical features can be enhanced by locally removing circular regions from the mask in steps of 1 nm^2. **b-i**, Top-view scanning electron micrographs of cavities with no global geometry-tuning, $\delta = 0$ nm. This work only considers LMC = 22 nm.

4 Quantifying the dimensions of fabricated devices

Figure S7**a-c** shows top-view scanning electron microscopy (SEM) images with < 100 pm pixels where the scan-direction is perpendicular to the bowtie (along the y-direction) to minimize drift errors in the measurement. The discontinuous outlines results as the dimensions are small compared to the resolution of the electron microscope. Figure S7**d-f** shows smoothed images to achieve a smooth outline of the top-view of the fabricated bowtie. We compute the width for each vertical line individually as shown in Figure S7**g** and **h** and fit a quadratic function around the center. A quadratic function is chosen since the topology optimization was tolerance-constrained to a void radius of curvature $r_v = 22$ nm. Although there are several nanometers of fluctuations in the widths in Fig. S7**g**, it is substantially lower than the fluctuations on either side in Fig. S7**a-c**, indicating that the scan fluctuations are correlated.

Figure S8 shows an array of different tilted views of different geometry-tuned devices from the same nominally identical copy (see optical spectra in Fig. 2 in the main text). This reveals that top-view SEM images alone are insufficient to characterize our cavities, as they do not reveal if the structure is correctly etched. We note that these measurements are consistent with the value of the device-layer thickness, $t = 240$ nm, which we measured with high precision using variable-angle ellipsometric spectroscopy before the topology optimization. These images also enable us to identify that the bowtie is etched during the first 8 cycles of the cyclic dry-etching process with each cycle etching ~ 30 nm, while 10 cycles are needed to etch the small air holes fully due to etch lag [31]. From Fig. S8**l** and **p** it can be seen that the process is free of notching [32]. From Fig. S8**g-h** and **k**, we estimate that the thickness of the bowtie is more narrow at the bottom, approximately 10 nm narrower, which indicates a sidewall negativity $\sim 1° \pm 0.5°$. Comparing Fig. S8**g-h** of nominally identical copy 5 to the tilted image in Fig. 1**e** in the main text of nominally identical copy 3 we note that the scallops penetrates the bowtie more places in copy 5 compared to copy 3. However, from the far-field measurements in Fig. 2 in the main text we observe a clear resonance for both cavities with resonant wavelength $\lambda_0 \approx 1440$ nm. Figure S8**h** shows that the scallops just penetrates the bowtie at the 6th scallop indicating a scallop depth of ~ 3 nm. Lastly, the 20 nm scale bars along y, and z are obtained by scaling the scale bar along x by $\cos(\theta)$ and $\sin(\theta)$, respectively, with θ the tilt angle.

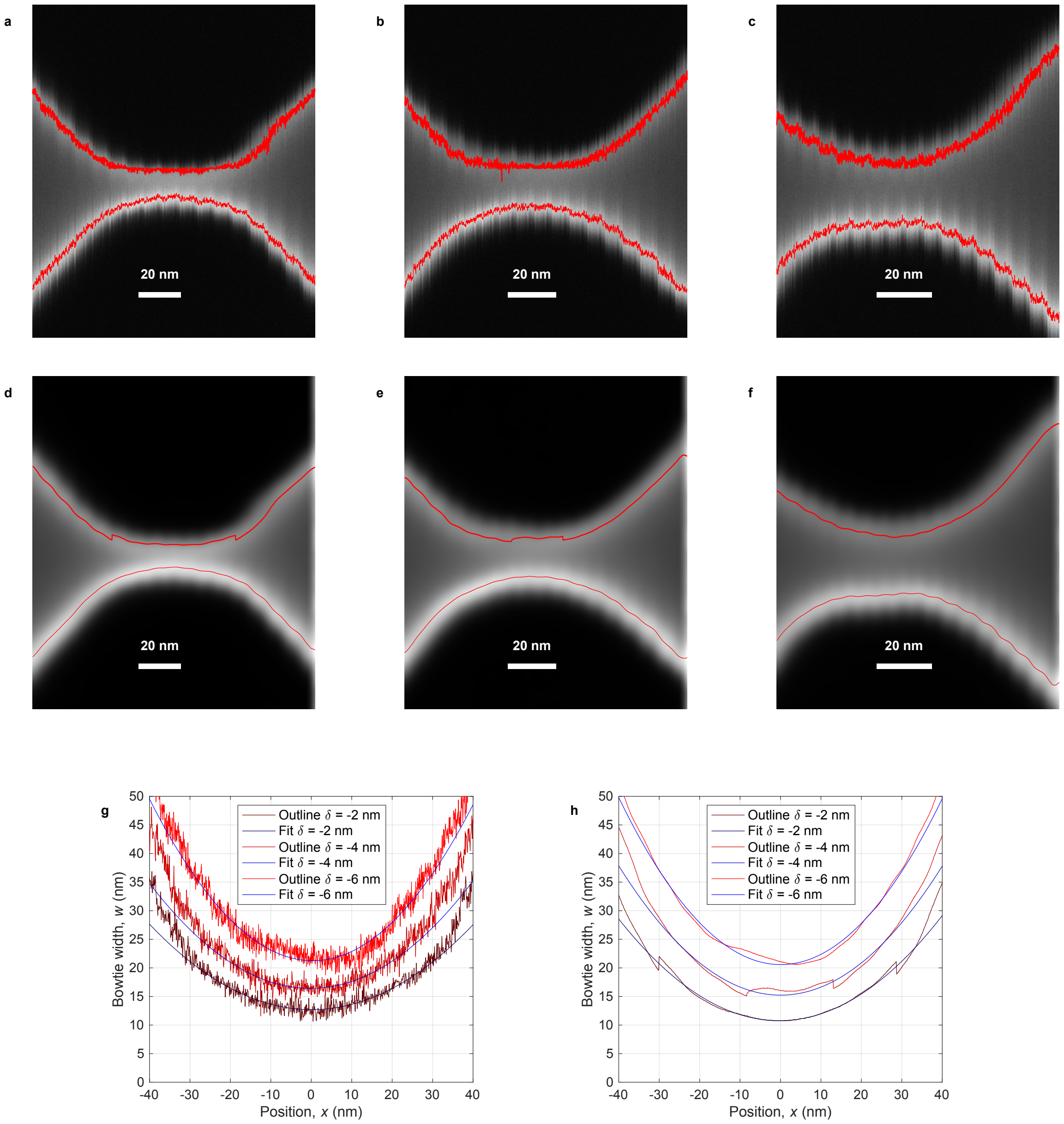

Fig. S7. Top-view scanning electron microscopy (SEM) images of dielectric bowties. a-c, Top-view SEM images acquired with a secondary electron detector under 25 keV excitation on nominally identical device copy 5 (with optical spectra shown in Fig. 2 in the main text) for global geometry-tuning, $\delta = -2$ nm, $\delta = -4$ nm, and $\delta = -6$ nm, respectively. The red lines traces the peak intensity in the SEM images and the distance between them measures the width. **d-f**, Smoothed SEMs from **a-c** using a Gaussian filter with 25 pixels variance. **g-h**, Widths obtained from **a-c** and **d-f**, respectively. The dark-red lines shows the measurements and the dark-blue lines a corresponding quadratic fit around the central part, yielding bowtie widths of 13 nm, 15 nm, and 21 nm, respectively for **d-f**, which combined with the small sidewall angle results in average widths (at the center of the membrane) of (8 ± 5) nm, (10 ± 5) nm, and (16 ± 5) nm, respectively.

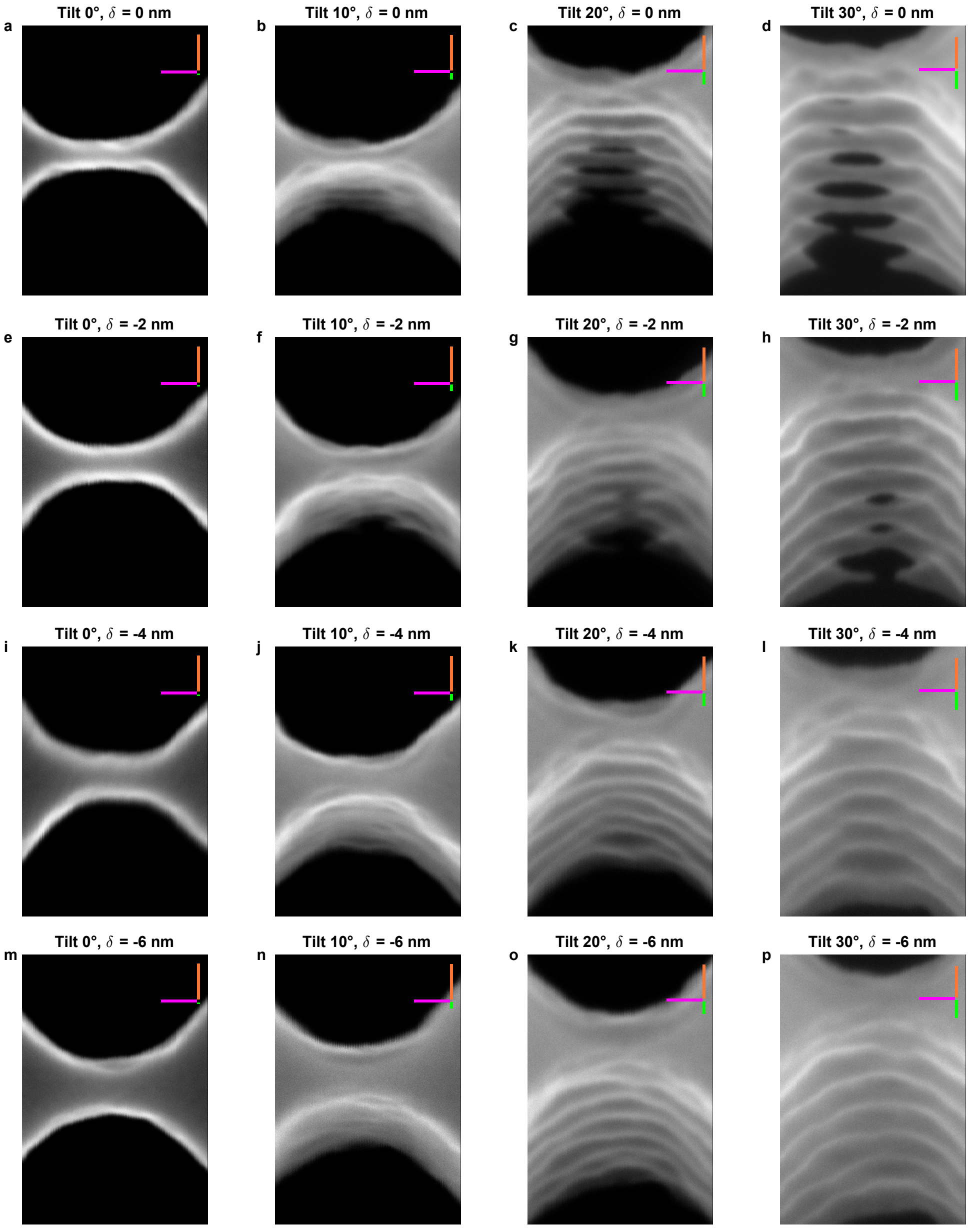

Fig. S8. Array of scanning electron microscopy (SEM) images of geometry-tuned cavities for different viewing angles. a-d, Shows 0°, 10°, 20°, and 30° tilted SEM images for global geometry-tuning $\delta = 0\,\mathrm{nm}$, where the bowtie is resolved but no resonance is measured in the far-field measurements. **e-h**, **i-l**, and **m-p**, Tilted images similar to **a-d** but for $\delta = -2\,\mathrm{nm}$, $\delta = -4\,\mathrm{nm}$, and $\delta = -6\,\mathrm{nm}$, respectively, showing devices where the optical mode is observed in far-field measurements (see Fig. 2 in the main text). All images are measured using a 19 keV electron beam with an in-lens detector collecting secondary electrons on nominally identical device copy 6. All scale bars are 20 nm, with the magenta, orange, and green scale bars showing the length along the x, y, and z directions, respectively. The green scale bars in **l** and **p** enables verification that the device layer is 240 nm thick, etched with 8 scallops that are each $\sim$ 30 nm tall.

5 Scattering scanning near-field optical microscopy measurements

We perform near-field measurements to experimentally verify the optical field confinement to a single hotspot in the middle of the cavities. To show this explicitly, this section presents near-field measurements on devices with different geometry-tunings, which results in different resonance wavelengths but essentially identical near-field maps. Figure S9 shows a schematic of the atomic force microscope (AFM) tip used for the near-field measurement. This gives reliable and detailed information about the near-field when scanning above the sample. However, when moving into a void feature, complex perturbations of the scattered light emerge and we discard data obtained in this regime for our final analysis. The excitation laser is s-polarized to minimize the excitation of the tip [33] and s-polarization of the scattered near-field is detected using a polarizer, as has been done in previous works [34, 35].

Figures S10**a** and **b** show two measurements of the near-field amplitude for a cavity with global geometry-tuning $\delta = -4$ nm recorded on resonance, $\lambda_0 = 1489.4$ nm, with two different spatial resolutions. This demonstrates that the field is confined in the center with excellent suppression of the background yielding strong correlation with the simulation as discussed in the main text. The contours of high field strengths shaped like the cavity holes on both sides of the center stem from the fact that the tip is moving inside the void features, which is consistent with the AFM measurement shown in Fig. S10**c**. The sharp silicon edges appear sloped as the tip shaft prevents the tip from going further down inside a hole. For clarity, Fig. S10**d** shows the same AFM map truncated to show only a thickness range of 40 nm. The same measurements and data analysis have been performed for the cavity with global geometry-tuning, $\delta = -6$ nm, shown in Figure S11, which yield similar results.

To map the resonance of the cavities in the near-field, we sweep the wavelength of the tunable excitation laser as discussed in Methods. A small map of the center of the cavity was acquired for wavelength steps of 0.5 nm, with an extra sampling spaced 0.2 nm around the resonance peak, in a range of 20 nm around the expected resonance from the far-field measurements. The results from these measurements are summarized in Fig. S12 and S13. Figures S12**a**-**j** present the spectra for the cavity with global geometry-tuning, $\delta = -4$ nm, along a line of varying x position and fixed y position. Most of the spectra have a resonance peak around 1489.4 nm.

The spectrum in the middle of the cavity is shown in Fig. S12**k**. We perform a fit with a Lorentzian in the frequency domain,

$$L(\omega) \propto \frac{\Gamma/2}{(\omega - \omega_0)^2 + (\Gamma/2)^2}, \tag{S1}$$

which gives a near-field quality factor, $Q_{\text{NF}} = \omega_0/\Gamma = 370 \pm 40$ around the resonant wavelength $\lambda_0 = (1489.4 \pm 0.1)$ nm. The near-field quality factor is lower than the quality factor measured in the far-field, which we attribute to losses induced by the presence of the tip [36].

Figure S12**l** shows an example of an AFM map acquired during the frequency sweep, here at the wavelength of $\lambda_0 = 1480$ nm. The blue arrow indicates the pixels selected to plot the spectrum in Fig. S12**b** and the green arrow shows the pixels for Fig. S12**j**. The red line indicates the x position of the cavity center. The same measurements and data treatment have been performed for the cavity of global geometry-tuning, $\delta = -6$ nm. These are shown in Fig. S13. We extract $\lambda_0 = (1522.9 \pm 0.1)$ nm from a Lorentzian fit and calculate the near-field quality factor $Q_{\text{NF}} = 510 \pm 50$.

Figure S14 shows a comparison between the dielectric bowtie cavities of different global geometry-tunings. Figures S14**a**-**b** are normalized to the maximum intensity in the maps, and Figs. S14**c**-**d** are normalized to the center with the air-regions blacked out to qualitatively assess the similarity between the measurements irregardless of the different bowtie widths as indicated by the tilted scanning electron microscopy (SEM) images in Figs. S14**e**-**f**.

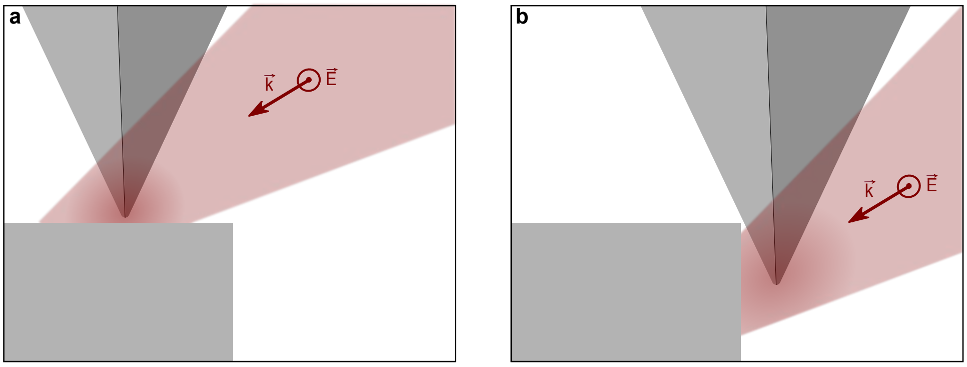

Fig. S9. Schematic of atomic force microscope (AFM) tip under optical illumination for scattering near-field optical microscopy. **a**, The AFM tip is made of silicon and has a nominal radius of $r \sim 10$ nm. It is operated in tapping mode and data is recorded when the tip is ~ 5 nm above the surface. The laser is s-polarized and has a diffraction-limited spot size of approximately 2 µm as indicated by the red fields and vectors. **b**, The measurements are governed by interactions between the sidewalls of the tip and the nanostructure when the tip is above void features of the geometry and we therefore discard these regions in our analysis of the measured mode.

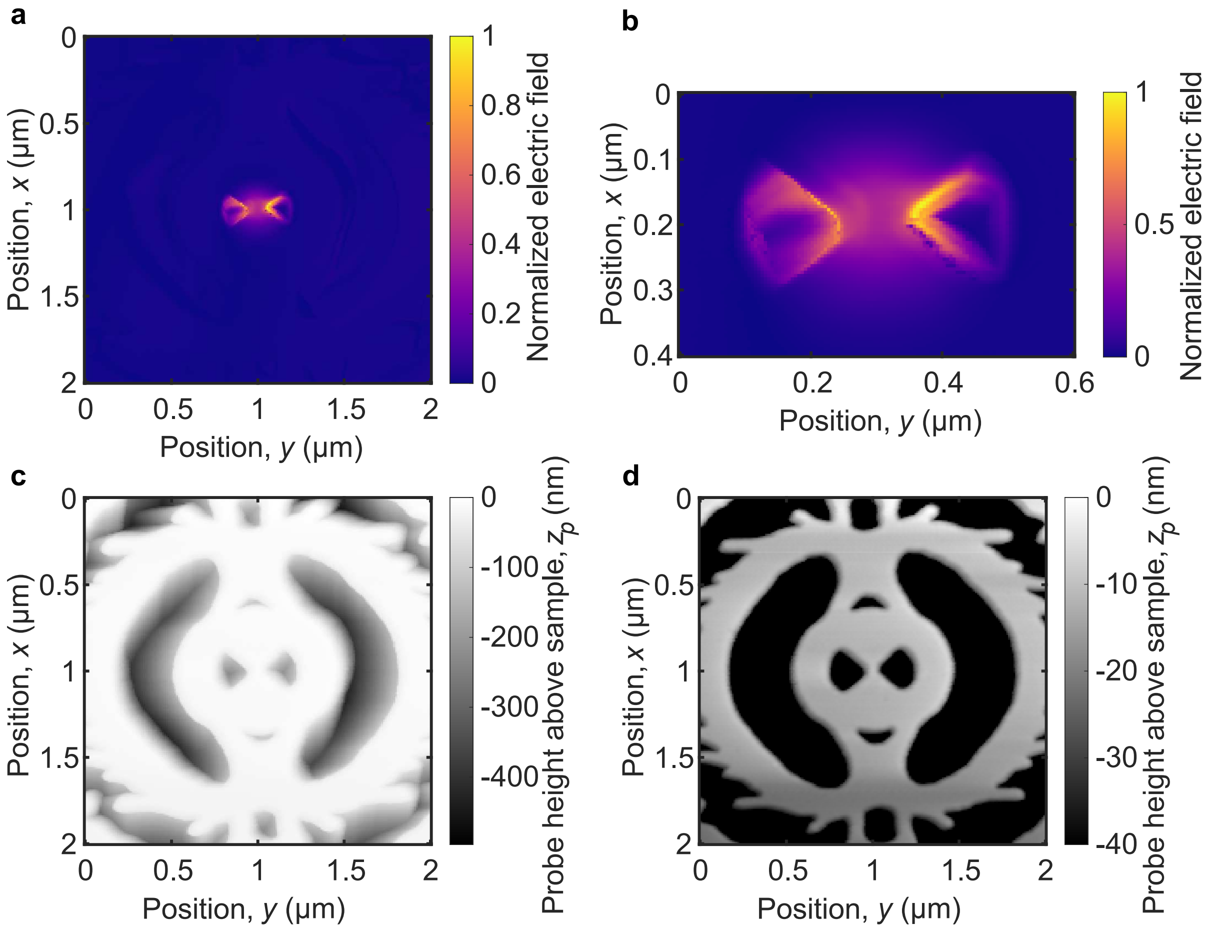

Fig. S10. Near-field mapping of a cavity with global geometry-tuning δ = −4 nm. **a-b**, Near-field amplitude, $|\boldsymbol{E}|$, of the cavity excited on-resonance, λ_0 = 1489.4 nm. **c-d**, Atomic force microscope (AFM) signal of the cavity, obtained from the same measurement, with **c** showing the full range where the tip moves through the air holes, and **d**, truncated at z_p = −40 nm to highlight the surface.

The location of the blacked-out region (i.e. the center of the map) is determined as the position yielding the best overlap between theory and experiment. The Bhattacharyya coefficient [37] for the overlap between the two maps in Figs. S14**a-b** is t = 0.985, and for the two blacked out maps in Figs. S14**c-d** it is t = 0.996. The overlap between the measurements for δ = −4 nm shown in Fig. S10**b** and the calculated mode is t = 0.984 when the calculated mode is convolved with a Gaussian instrument function with σ = (37 ± 5) nm, consistent with the results of the main text.

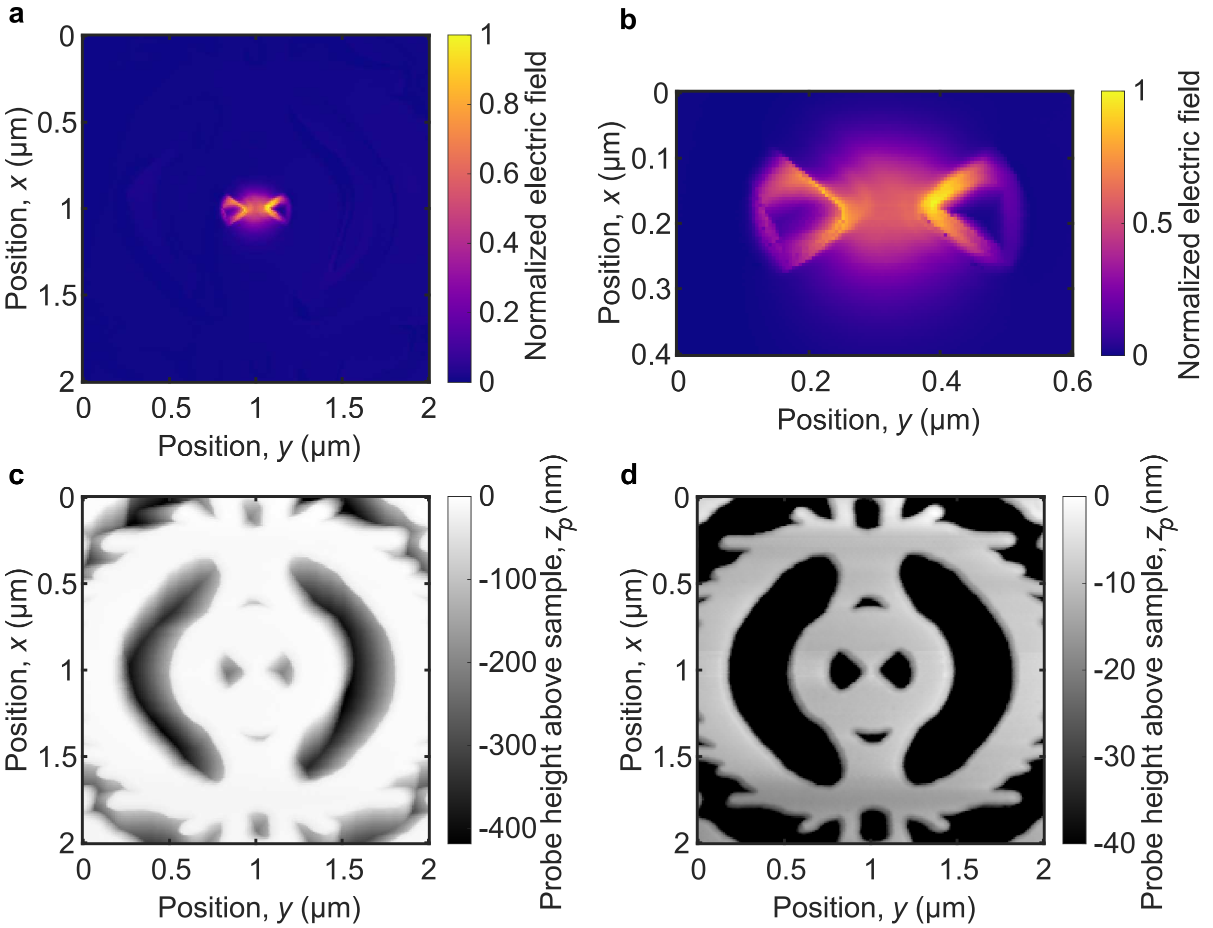

Fig. S11. Near-field mapping of a cavity with global geometry-tuning $\delta = -6$ nm. **a-b**, Near-field amplitude, $|\boldsymbol{E}|$, of the cavity excited on-resonance, $\lambda_0 = 1522.9$ nm. **c-d**, Atomic force microscope (AFM) signal of the cavity, obtained from the same measurement, with **c** showing the full range where the tip moves through the air-holes, and **d**, truncated at $z_p = -40$ nm to highlight the surface.

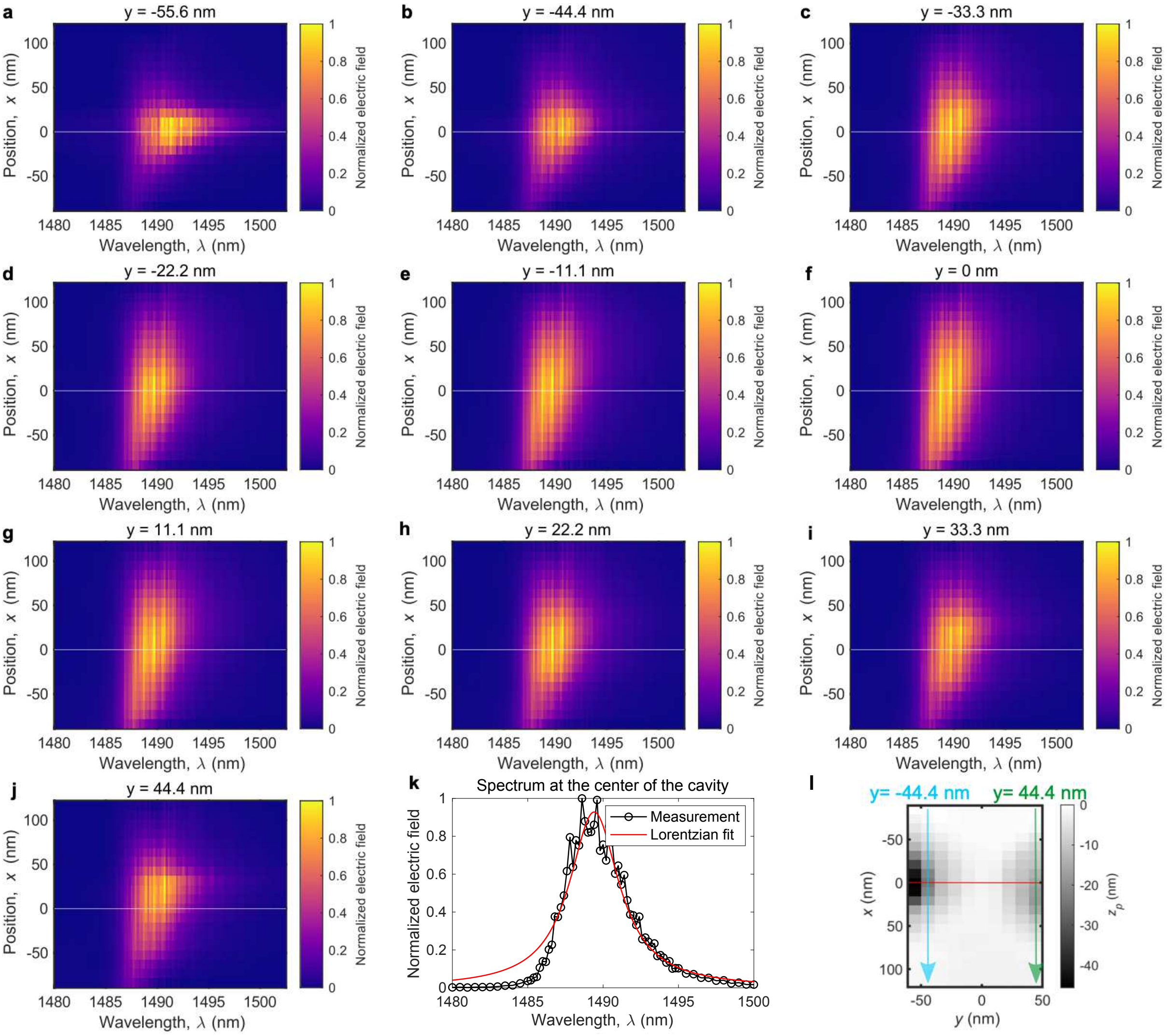

Fig. S12. Near-field spectrum of a cavity with global geometry-tuning $\delta = -4\,\mathrm{nm}$. a-j, s-SNOM spectra at different wavelengths for fixed y positions, scanning along the bowtie. The white line indicates the x position of the cavity center. **k**, Spectrum at the cavity center with a Lorentzian fit. **l**, Example of an AFM map obtained during the s-SNOM measurements. The red line indicates the x position of the cavity center.

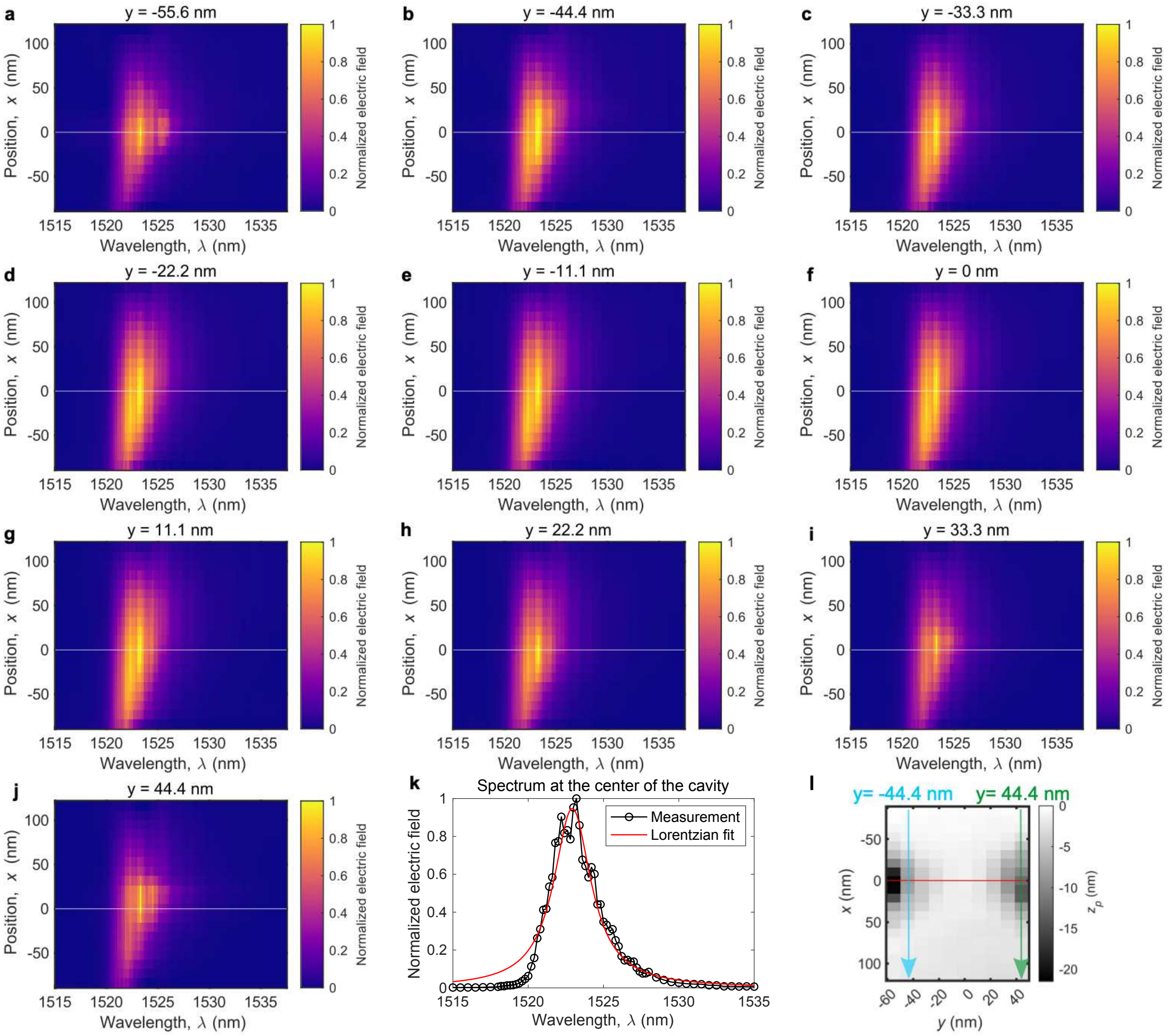

Fig. S13. Near-field spectrum of a cavity with global geometry-tuning $\delta = -6$ nm. a-j, s-SNOM spectra at different wavelengths for fixed y positions, scanning along the bowtie. The white line indicates the x position of the cavity center. **k**, Spectrum at the cavity center with Lorentzian fit. **l**, Example of an AFM map taken for the spectra measurements. The red line indicates the x position of the cavity center.

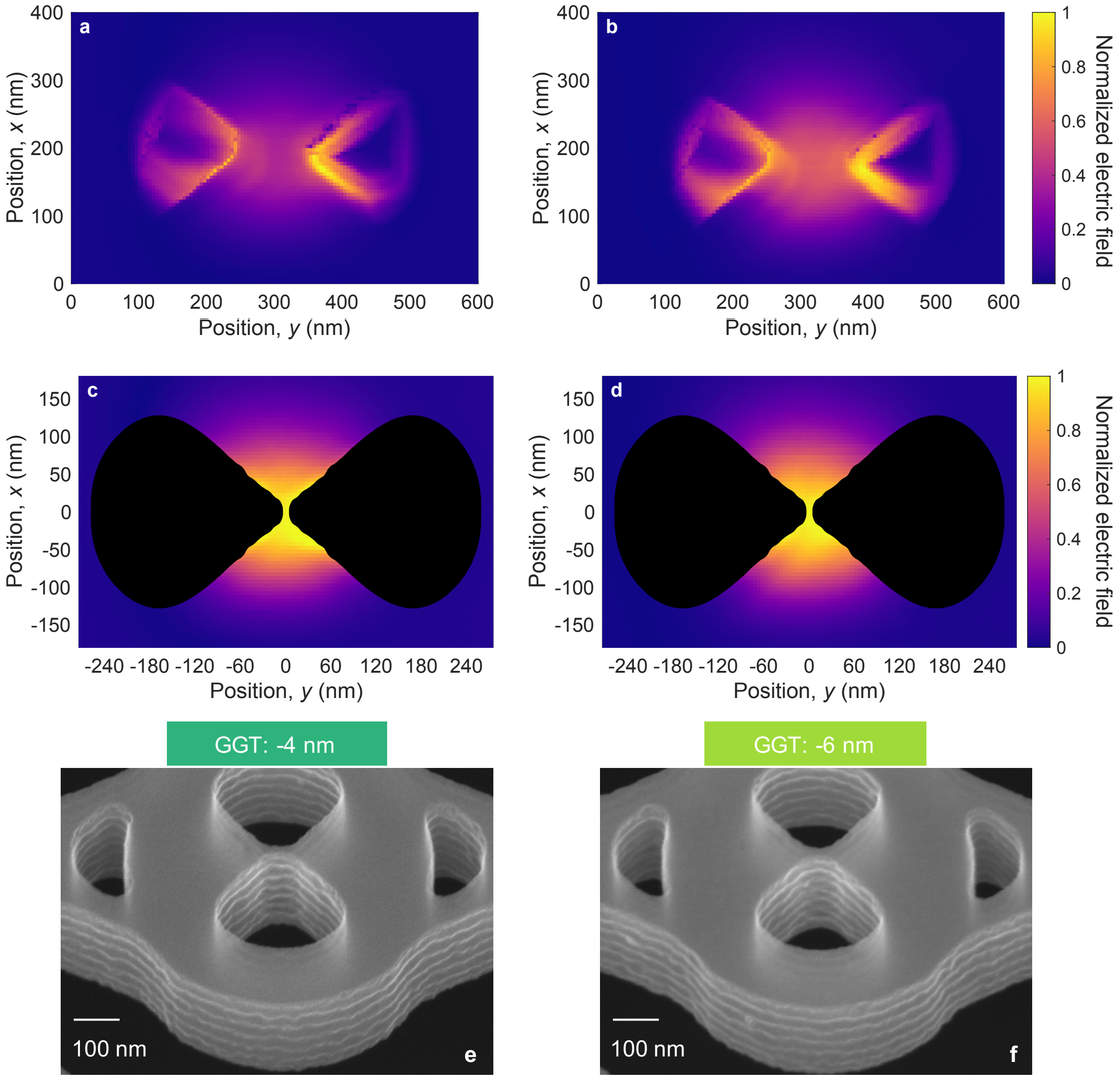

Fig. S14. Comparison of near-field measurements of cavities with different global geometry-tuning, δ. a-b, Normalized electric field amplitude obtained by s-SNOM measurements of DBCs with $\delta = -4$ nm and $\delta = -6$ nm, respectively. Each map is normalized to the maximum measured. **c-d**, Same measurements as **a-b** but centered and with void features blacked out and normalized to the value at the center, emphasizing that the measurements are near identical for different bowtie widths. **e-f**, Scanning electron micrographs of the cavity bowties measured with s-SNOM showing the difference in bowtie widths as discussed in the main text.

6 Overview of experimental data in this work

The present paper reports experimental results of a sample with 336 dielectric bowtie cavities, split across six nominally identical copies of 56 different cavities. Each of the 56 cavities are different due to systematic modifications of the exposure mask according to the following procedure: First, the mask is modified by a local mask correction (LMC) around the central bowtie, see Fig. S6, and second, the mask is tuned by a global geometry-tuning (δ), see Fig. S5. All figures, with the exception of Fig. S6, report data of cavities with LMC = 22 nm. Table S1 lists all figures that present experimental data as well as the corresponding device (copy and global geometry-tuning) used to produce it. The near-field optical measurements are not performed on devices with $\delta = -2$ nm, corresponding to a mean bowtie bridge width, $w = (8 \pm 5)$ nm, since their resonance at $\lambda_0 \sim 1440$ nm lies outside the range of the s-SNOM excitation laser, see Methods.

Figure	Device description
Fig. 1c	Copy 3, $\delta = -2$ nm.
Fig. 1e	Copy 3, $\delta = -2$ nm.
Fig. 1f	Copy 3, $\delta = -4$ nm.
Fig. 1g	Copy 3, $\delta = -6$ nm.
Fig. 2a	Copy 5, $\delta = -6$ nm.
Fig. 2b	Copy {1-6}, $\delta = -2$ nm.
Fig. 2c	Copy {1-6}, $\delta = -4$ nm.
Fig. 2d	Copy {1-6}, $\delta = -6$ nm.
Fig. 2e	Mean and standard deviation across all 18 cavities.
Fig. 3a to d	Copy 3, $\delta = -4$ nm.
Fig. S5**a-b**	Precursor sample 1 to quantify the radius of curvature.
Fig. S5**c**	Precursor sample 2 to quantify dry-etch performance.
Fig. S5**d-e**	Copy 3, $\delta = -6$ nm.
Fig. S6**b**,**f**	Copy 3, $\delta = 0$ nm.
Fig. S6**c**,**g**	Copy 3, $\delta = -2$ nm.
Fig. S6**d**,**h**	Copy 3, $\delta = -4$ nm.
Fig. S6**e**,**i**	Copy 3, $\delta = -6$ nm.
Fig. S7**b-i**	Copy 1, $\delta = 0$ nm, all LMC.
Fig. S8**a**,**d**	Copy 6, $\delta = -2$ nm.
Fig. S8**b**,**e**	Copy 6, $\delta = -4$ nm.
Fig. S8**c**,**f**	Copy 6, $\delta = -6$ nm.
Fig. S8**g-h**	Copy 6, all δ.
Fig. S9**a-d**	Copy 6, $\delta = 0$ nm.
Fig. S9**e-h**	Copy 6, $\delta = -2$ nm.
Fig. S9**i-l**	Copy 6, $\delta = -4$ nm.
Fig. S9**m-p**	Copy 6, $\delta = -6$ nm.
Fig. S11**m-p**	Copy 3, $\delta = -4$ nm.
Fig. S12**m-p**	Copy 3, $\delta = -6$ nm.
Fig. S13**m-p**	Copy 3, $\delta = -4$ nm.
Fig. S14**m-p**	Copy 3, $\delta = -6$ nm.
Fig. S15**a**,**c**,**e**	Copy 3, $\delta = -4$ nm.
Fig. S15**b**,**d**,**f**	Copy 3, $\delta = -6$ nm.

Table S1 Overview of data, figures, and results with corresponding devices. All data except Fig. S6 reports on devices with local mask correction, LMC = 22 nm.

Supplementary References

[1] P. Lodahl, S. Mahmoodian, and S. Stobbe, “Interfacing single photons and single quantum dots with photonic nanostructures”, Rev. Mod. Phys. **87**, 347–400 (2015).

[2] P. T. Kristensen, C. Van Vlack, and S. Hughes, “Generalized effective mode volume for leaky optical cavities”, Opt. Lett. **37**, 1649–1651 (2012).

[3] S. Sanders and A. Manjavacas, “Analysis of the limits of the local density of photonic states near nanostructures”, ACS Photonics **5**, 2437–2445 (2018).

[4] X. Liang and S. G. Johnson, “Formulation for scalable optimization of microcavities via the frequency-averaged local density of states”, Opt. Express **21**, 30812–30841 (2013).

[5] F. Wang, R. E. Christiansen, Y. Yu, J. Mørk, and O. Sigmund, “Maximizing the quality factor to mode volume ratio for ultra-small photonic crystal cavities”, Appl. Phys. Lett. **113**, 241101 (2018).

[6] P. T. Kristensen, R.-C. Ge, and S. Hughes, “Normalization of quasinormal modes in leaky optical cavities and plasmonic resonators”, Phys. Rev. A **92**, 053810 (2015).

[7] P. T. Kristensen, K. Herrmann, F. Intravaia, and K. Busch, “Modeling electromagnetic resonators using quasinormal modes”, Adv. Opt. Photon. **12**, 612–708 (2020).

[8] J.-M. Jin, *The finite element method in electromagnetics*, 3rd ed. (N.J., 2015).

[9] M. Zhou, B. S. Lazarov, F. Wang, and O. Sigmund, “Minimum length scale in topology optimization by geometric constraints”, Comput. Methods Appl. Mech. Eng. **293**, 266–282 (2015).

[10] A. Sommerfeld, *Mathematical theory of diffraction*, 1st ed., Translated by R. J. Nagem, M. Zampolli, G. Sandri. (Original work published Math. Ann. **47**, 317–374 (1896).) (Boston, MA, 2004).

[11] L. D. Landau, E. M. Lifshitz, and L. P. Pitaevskii, *Electrodynamics of continuous media*, 2nd ed., Translated by J. B. Sykes, J. S. Bell, M. J. Kearsley. (Oxford, 1984), (Original work published 1982).

[12] J. D. Jackson, *Classical electrodynamics*, 3rd (N.J., 1999).

[13] J. Andersen and V. Solodukhov, “Field behavior near a dielectric wedge”, IEEE Trans. Antennas Propag. **26**, 598–602 (1978).

[14] J. Van Bladel, “Field singularities at the tip of a dielectric cone”, IEEE Trans. Antennas Propag. **33**, 893–895 (1985).

[15] N. A. Mortensen, S. Raza, M. Wubs, T. Søndergaard, and S. I. Bozhevolnyi, “A generalized non-local optical response theory for plasmonic nanostructures”, Nat. Commun. **5**, 3809 (2014).

[16] H. Choi, M. Heuck, and D. Englund, “Self-similar nanocavity design with ultrasmall mode volume for single-photon nonlinearities”, Phys. Rev. Lett. **118**, 223605 (2017).

[17] M. Albrechtsen, B. Vosoughi Lahijani, and S. Stobbe, “Two regimes of confinement in photonic nanocavities: bulk confinement versus lightning rods”, Opt. Express **30**, 15458–15469 (2022).

[18] A. Säynätjoki, B. Bai, A. Tervonen, J. Turunen, and S. Honkanen, “Enhanced vertical confinement in angled-wall slot waveguides”, Opt. Rev. **17**, 181–186 (2010).

[19] V. R. Almeida, Q. Xu, C. A. Barrios, and M. Lipson, “Guiding and confining light in void nanostructure”, Opt. Lett. **29**, 1209–1211 (2004).

[20] Q. Zhao, L. Zhang, and O. D. Miller, “Minimum dielectric-resonator mode volumes”, arxiv:2008.13241 (2020).

[21] J. T. Robinson, C. Manolatou, L. Chen, and M. Lipson, “Ultrasmall mode volumes in dielectric optical microcavities”, Phys. Rev. Lett. **95**, 143901 (2005).

[22] A. Gondarenko et al., “Spontaneous emergence of periodic patterns in a biologically inspired simulation of photonic structures”, Phys. Rev. Lett. **96**, 143904 (2006).

[23] A. Gondarenko and M. Lipson, “Low modal volume dipole-like dielectric slab resonator”, Opt. Express **16**, 17689–17694 (2008).

[24] K. Schneider and P. Seidler, “Strong optomechanical coupling in a slotted photonic crystal nanobeam cavity with an ultrahigh quality factor-to-mode volume ratio”, Opt. Express **24**, 13850–13865 (2016).

[25] S. Hu and S. M. Weiss, “Design of photonic crystal cavities for extreme light concentration”, ACS Photonics **3**, 1647–1653 (2016).

[26] S. Mignuzzi et al., “Nanoscale design of the local density of optical states”, Nano Lett. **19**, 1613–1617 (2019).

[27] H. Sekoguchi, Y. Takahashi, T. Asano, and S. Noda, “Photonic crystal nanocavity with a Q-factor of ~9 million”, Opt. Express **22**, 916–924 (2014).

[28] V. T. H. Nguyen et al., “The CORE sequence: a nanoscale fluorocarbon-free silicon plasma etch process based on SF_6/O_2 cycles with excellent 3D profile control at room temperature”, ECS J. Solid State Sci. Technol. **9**, 024002 (2020).

[29] V. T. H. Nguyen et al., “Ultrahigh aspect ratio etching of silicon in SF_6-O_2 plasma: the clear-oxidize-remove-etch (CORE) sequence and chromium mask”, J. Vac. Sci. Technol. A **38**, 053002 (2020).

[30] V. T. H. Nguyen et al., “Cr and CrO_x etching using SF_6 and o_2 plasma”, J. Vac. Sci. Technol. B **39**, 032201 (2021).

[31] H. Jansen et al., “BSM 7: RIE lag in high aspect ratio trench etching of silicon”, Microelectron. Eng. **35**, 45–50 (1997).
[32] G. S. Hwang and K. P. Giapis, “On the origin of the notching effect during etching in uniform high density plasmas”, J. Vac. Sci. Technol. B **15**, 70–87 (1997).
[33] L. Novotny and B. Hecht, *Principles of nano-optics*, 2nd ed. (2012).
[34] M. Schnell, A. Garcia-Etxarri, J. Alkorta, J. Aizpurua, and R. Hillenbrand, “Phase-resolved mapping of the near-field vector and polarization state in nanoscale antenna gaps”, Nano Lett. **10**, 3524–3528 (2010).
[35] Z. H. Kim and S. R. Leone, “Polarization-selective mapping of near-field intensity and phase around gold nanoparticles using apertureless near-field microscopy”, Opt. Express **16**, 1733–1741 (2008).
[36] S. Götzinger, O. Benson, and V. Sandoghdar, “Towards controlled coupling between a high-Q whispering-gallery mode and a single nanoparticle”, Appl. Phys. B. **73**, 825–828 (2001).
[37] A. Bhattacharyya, “On a measure of divergence between two statistical populations defined by their probability distributions”, Bull. Calcutta Math. Soc. **35**, 99–109 (1943).
